# SpaGIC: graph-informed clustering in spatial transcriptomics via self-supervised contrastive learning

**DOI:** 10.1093/bib/bbae578

**Published:** 2024-11-14

**Authors:** Wei Liu, Bo Wang, Yuting Bai, Xiao Liang, Li Xue, Jiawei Luo

**Affiliations:** College of Computer Science and Electronic Engineering, Hunan University, Changsha 410083, China; College of Computer Science and Electronic Engineering, Hunan University, Changsha 410083, China; College of Computer Science and Electronic Engineering, Hunan University, Changsha 410083, China; College of Computer Science and Electronic Engineering, Hunan University, Changsha 410083, China; College of Computer Science and Electronic Engineering, Hunan University, Changsha 410083, China; College of Computer Science and Electronic Engineering, Hunan University, Changsha 410083, China

**Keywords:** spatial transcriptomics, spatial domain identification, graph convolutional networks, self-supervised contrastive learning

## Abstract

Spatial transcriptomics technologies enable the generation of gene expression profiles while preserving spatial context, providing the potential for in-depth understanding of spatial-specific tissue heterogeneity. Leveraging gene and spatial data effectively is fundamental to accurately identifying spatial domains in spatial transcriptomics analysis. However, many existing methods have not yet fully exploited the local neighborhood details within spatial information. To address this issue, we introduce SpaGIC, a novel graph-based deep learning framework integrating graph convolutional networks and self-supervised contrastive learning techniques. SpaGIC learns meaningful latent embeddings of spots by maximizing both edge-wise and local neighborhood-wise mutual information of graph structures, as well as minimizing the embedding distance between spatially adjacent spots. We evaluated SpaGIC on seven spatial transcriptomics datasets across various technology platforms. The experimental results demonstrated that SpaGIC consistently outperformed existing state-of-the-art methods in several tasks, such as spatial domain identification, data denoising, visualization, and trajectory inference. Additionally, SpaGIC is capable of performing joint analyses of multiple slices, further underscoring its versatility and effectiveness in spatial transcriptomics research.

## Introduction

Complex tissues are composed of diverse cells, and the relative location of transcriptional expression within tissues is critical for understanding their biological functions and inferring cell–cell communications [1, 2]. Advanced spatial transcriptomics (ST) technologies have enabled genome-wide analysis at various resolutions, including multicellular, single-cell, or even subcellular levels, while preserving spatial information [3, 4]. Existing ST technologies can be broadly classified into two categories. One is in situ capturing-based technologies, such as Slide-seq [5], Slide-seqV2 [6], 10x Visium [7], and Stereo-seq [8], which measure the expression of thousands of genes across the genome of capture sites. The other category includes in situ hybridization or sequencing-based technologies like seqFISH [9, 10], MERFISH [11, 12], osmFISH [13], and STARmap [14], which measure the expression of hundreds to thousands of genes in cells with high resolution and accuracy. These ST technologies play a crucial role in enabling a comprehensive comprehension of the emerging properties and pathology of tissues, as well as revealing the heterogeneity of complex tissues. However, significant differences exist between datasets obtained from different technologies, posing challenges in developing a universal computational framework for ST analysis.

Identifying spatial domains, which are regions displaying spatial consistency in gene expression and histology, is a crucial task in ST analysis [15]. Previous studies have applied various clustering methods to spatial transcriptomic data analysis, including those specifically developed for scRNA-seq data, such as scGAC [16] and scTPC [17], as well as traditional clustering methods like k-means and Louvain [18]. However, these methods focus solely on gene expression data, disregarding valuable spatial information and leading to inaccuracies in identifying spatial domains. To address this limitation and better reveal the spatial distribution of cells in tissues, several spatial clustering methods have been emerged. For example, BayesSpace [19] and Giotto [20] apply Bayesian and hidden Markov random field models, respectively, to detect spatial domains. While effective in capturing spatial information, these statistics-based methods fall short in capturing the nonlinear features of gene expression, thereby limiting their performance. In contrast, deep learning methods have developed to overcome this limitation, with many leveraging graph neural networks as the fundamental framework. For instance, STAGATE [15] utilizes an adaptive graph attention auto-encoder to integrate spatial information and gene expression data. DeepST [21] and SEDR [22] employ variational graph auto-encoders to learn the latent representation of spots for downstream analysis. Similarly, SpaGCN [23], CCST [24], GraphST [25], and Spatial-MGCN [26] use graph convolutional networks (GCNs) to aggregate neighbor information for effectively identifying spatial domains. Moreover, SpaGCN and DeepST implement data augmentation by using morphological features extracted from histological images through pre-trained models. However, this augmentation, while offering some performance improvement, greatly reduces their execution efficiency. CCST and GraphST adopt the Deep Graph Infomax (DGI) [27] and DGI-like models, respectively, to enhance spots representation learning but are limited in considering only global graph summaries. Consequently, they are unable to incorporate the rich fine-grained semantic and structural information contained in local neighborhoods into spots representation. Notably, only a subset of these methods, such as STAGATE, GraphST, and SEDR, can jointly analyze multiple tissue slices while correcting batch effects.

In this work, we propose a novel self-supervised contrastive learning framework, SpaGIC, for graph-informed clustering in ST. The framework integrates gene expression data and spatial information through a GCN-based auto-encoder, learning discriminative spots representation. To verify the effectiveness of SpaGIC, we performed multiple ST analysis tasks and compared it with existing methods on seven ST datasets generated by different technology platforms (e.g. 10x Visium, Stereo-seq, Slide-seqV2, STARmap, and osmFISH). The experimental results demonstrated that SpaGIC achieved competitive performance over existing state-of-the-art methods in spatial domain identification, data denoising, visualization, and trajectory inference. More importantly, SpaGIC can be combined with Harmony [28] to perform multi-slice joint analysis while correcting batch effects.

## Materials and methods

### Overview of SpaGIC

**Figure 1:**
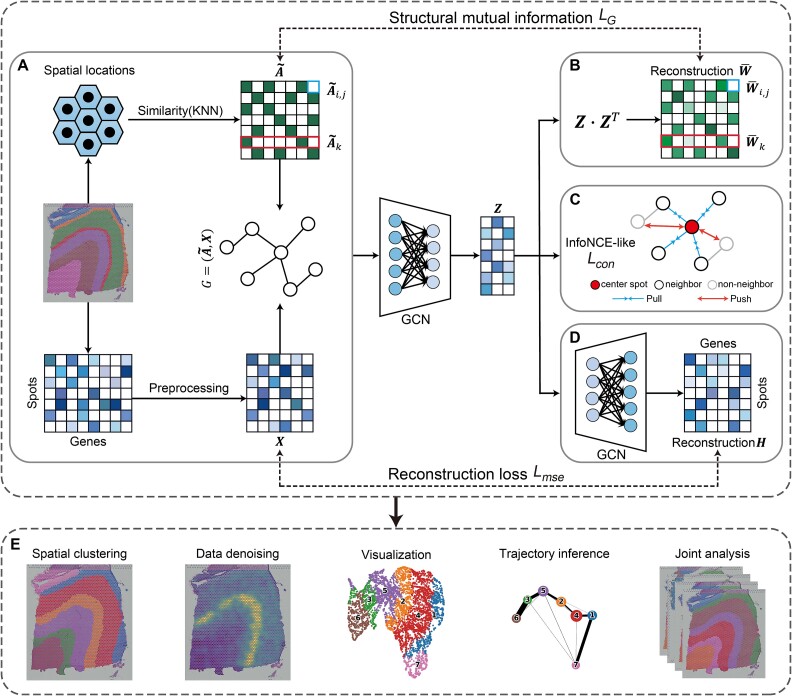
The overall framework of SpaGIC. (**A**) SpaGIC first constructs a feature graph $G$, where the node connection relationships are calculated by KNN based on spatial location similarity, and node features are derived from preprocessed gene expression data. Next, SpaGIC employs a GCN-based auto-encoder to learn the node latent embeddings $\boldsymbol{Z}$. (**B**) These latent representations are then used to reconstruct the connection weights between nodes and establish mutual information constraints on the graph structures. (**C**) Calculating an InfoNCE-like loss based on the learned embeddings introduces a contrastive learning constraint. (**D**) A decoder reverses the embeddings back into the original feature space to reconstruct the gene expression matrix $\boldsymbol{H}$. (**E**) The output of SpaGIC can be applied to various ST downstream analysis tasks, such as spatial clustering, data denoising, visualization, trajectory inference, and multi-slice joint analysis.

SpaGIC is a novel graph-based self-supervised contrastive learning framework for effective ST analysis, as shown in Fig. 1. In the workflow of SpaGIC, a GCN-based auto-encoder is utilized to learn spots representation by iteratively aggregating information from neighboring nodes. Initially, the framework employs the KNN algorithm to construct an adjacency graph according to spots spatial location, and takes preprocessed gene expression data as nodes feature (Fig. 1A). Based on the embeddings, SpaGIC reconstructs the adjacency to enhance spots representation through maximizing graph structural mutual information both edge-wise and local neighborhood-wise in a self-supervised manner (Fig. 1B). Concurrently, an InfoNCE-like contrastive learning loss is integrated into the model training to preserve the spatial neighbor information by grouping spatially adjacent spots and separating spatially non-adjacent spots in latent space (Fig. 1C). Furthermore, to fully exploit the gene expression profiles, SpaGIC reconstructs the raw gene expression matrix from latent embeddings using a decoder, while adhering to the constraint of MSE (Mean Squared Error) loss (Fig. 1D). The resulting output from SpaGIC is versatile and can be applied across a range of downstream ST analysis tasks, such as spatial domain identification, data denoising, visualization, trajectory inference, and multi-slice joint analysis (Fig. 1E).

### Data description and preprocessing

Multiple publicly available datasets from different platforms, including 10x Visium, Slide-seqV2, Stereo-seq, STARmap, osmFISH, and seqFISH (see Supplementary Table S1 for details), were utilized to evaluate the performance of SpaGIC. Specifically, the LIBD human dorsolateral prefrontal cortex (DLPFC) dataset [29] comprises 12 slices from three individuals obtained with 10x Visium. Each slice contains 3498 to 4789 spots, captures 33 538 genes, and is manually annotated to include five to seven regions, namely the DLPFC layers and white matter (WM). The 10x Visium human breast cancer (HBC) dataset [30], with 3798 cells and 36 601 genes, is manually annotated by SEDR [22] based on H*&*E staining and pathological features. The anterior section of the 10x Visium mouse brain, with 2695 spots and 32 285 genes, is manually annotated by GraphST [25] into 52 domains. The mouse olfactory bulb (MOB) datasets obtained through Slide-seqV2 [6] and Stereo-seq [8] contain 21 724 and 19 109 spots, respectively. The mouse visual cortex (MVC) dataset [14], with 1207 cells and 1020 genes, is acquired using STARmap. Lastly, the mouse somatosensory cortex (MSC) dataset [13], with 6471 cells and 33 genes, is acquired using osmFISH.

For all datasets, genes expressed in fewer than three spots are first filtered. The Scanpy package [31] is then employed to identify highly variable genes (HVGs). Following this, the raw gene expression counts are normalized based on library size, log-transformed, and scaled. If the number of filtered genes is greater than 3000, the top 3000 HVGs are chosen as inputs for the SpaGIC model; otherwise, all filtered genes are used as inputs for the SpaGIC model.

### Spatial graph construction

The biological function of a cell is closely related to its surroundings cells, highlighting the importance of considering spatial relationships in analyzing cellular interactions. To fully utilize the spatial information of spots, a spatial graph $G=(\boldsymbol{A},\boldsymbol{X})$ is constructed using the $k$-nearest neighbor (KNN) method based on spatial similarity. In this graph $G$, $\boldsymbol{A}\in R^{N\times N}$ represents the adjacency matrix of $N$ spots and $\boldsymbol{X}\in R^{N\times M}$ is the preprocessed gene expression data with $M$ denoting the number of filtered genes. To determine the spatial similarity $\boldsymbol{S}_{ij}$ between spots $i$ and $j$, the Gaussian kernel function is applied to the spatial information as follows:


(1) \begin{align*}& \boldsymbol{S}_{ij}=\exp\left(\frac{\left\|p_{i}-p_{j}\right\|_{2}^{2}}{-2l^{2}}\right)\end{align*}

where $p_{i}$ and $p_{j}$ denote the spatial coordinates of spots $i$ and $j$. The $l$ is a hyperparameter that controls the rate at which the similarity between spots diminishes with distance. For a given spot $i$, the top-$k$ nearest spots are selected as its neighbors, thereby setting $\boldsymbol{A}_{ij}=\boldsymbol{A}_{ji}=1$, otherwise 0. Additionally, a self-loop is incorporated for each spot, resulting in $\widetilde{\boldsymbol{A}}=\boldsymbol{A}+\boldsymbol{I}$. In our experiment, we have empirically determined an appropriate value for $k$ to ensure that each spot, on average, is linked to between 5 and 15 neighboring spots.

### GCN-based auto-encoder for latent representation learning

GCN [32] can fully utilize the adjacency relationships between nodes in the graph to aggregate information from neighbors. SpaGIC adopts a GCN-based auto-encoder to learn the latent representation of spots.

The encoder uses the spatial graph $G=(\widetilde{\boldsymbol{A}},\boldsymbol{X})$ as input to iteratively aggregate information from neighboring nodes for learning discriminative spots representation $\boldsymbol{Z}$. Formally, the output of the $l$th layer in the encoder can be formulated as follows:


(2) \begin{align*}& \boldsymbol{Z}^{l}=\sigma\left(\boldsymbol{D}^{-\frac{1}{2}} \widetilde{\boldsymbol{A}} \boldsymbol{D}^{-\frac{1}{2}} Z^{l-1} \boldsymbol{W}^{l-1}\right)\end{align*}

where $\sigma $ is the nonlinear activation function and the default is Relu (Rectified Linear Unit). $\boldsymbol{D}$ is the diagonal degree matrix of $\widetilde{\boldsymbol{A}}$ and $\boldsymbol{D}^{-\frac{1}{2}} \widetilde{\boldsymbol{A}} \boldsymbol{D}^{-\frac{1}{2}}$ represents the normalized adjacent matrix. The initial $\boldsymbol{Z}^{0}=\boldsymbol{X}$ and $\boldsymbol{W}^{l-1}$ is the trainable weight matrix of the $l$th layer. The encoder consists of two GCN layers containing 512 and 64 neurons, respectively.

The decoder adopts a symmetrical structure with the encoder and reverses the latent representation $\boldsymbol{Z}$ back to reconstructed gene expression profiles. Specifically, the output of the $m$th layer in the decoder can be formulated as follows:


(3) \begin{align*}& \boldsymbol{H}^{m}=\sigma\left(\boldsymbol{D}^{-\frac{1}{2}} \widetilde{\boldsymbol{A}} \boldsymbol{D}^{-\frac{1}{2}} \boldsymbol{H}^{m-1} \widehat{\boldsymbol{W}}^{m-1}\right)\end{align*}

where the initial $\boldsymbol{H}^{0}=\boldsymbol{Z}$ and $\widehat{\boldsymbol{W}}^{m-1}$ is the trainable weight matrix of the $m$th layer. The last layer of the decoder outputs the reconstructed gene expression matrix $\boldsymbol{H}$.

To fully capture valuable information within gene expression profiles, the reconstruction loss of gene expression is applied to promote model training as follows:


(4) \begin{align*}& L_{mse}=\frac{1}{N}\sum_{i}^{N}\parallel \boldsymbol{X}_{i}-\boldsymbol{H}_{i}\parallel^{2}\end{align*}

where $\boldsymbol{X}_{i}$ and $\boldsymbol{H}_{i}$ are the raw preprocessed gene expression and reconstructed gene expression for spot $i$, respectively.

### Self-supervised contrast learning constraints

Inspired by DGSI (Deep Graph Structural Infomax) [33], we introduce constraints based on graph structures to facilitate the learning of spots representation. These constraints aim to maximize mutual information both edge-wise and local neighborhood-wise in a self-supervised manner. Specifically, in the latent embedding space, the expected edge connection weight between spots $i$ and $j$ is calculated as follows:


(5) \begin{align*}& \overline{\boldsymbol{W}}_{ij}=\boldsymbol{Z}_{i}\cdot \boldsymbol{Z}_{j}^{T}\end{align*}

where $\boldsymbol{Z}_{i}$, $\boldsymbol{Z}_{j}$ are the latent representation of spots $i$ and $j$. It should be noted that when $\overline{\boldsymbol{W}}$ is used to calculate edge-wise and local neighborhood-wise mutual information constraints, sigmoid and softmax processing are performed respectively.

To be more specific, minimizing the binary cross entropy (BCE) loss between the input adjacency matrix $\widetilde{\boldsymbol{A}}$ and the reconstructed edge weight matrix $\overline{\boldsymbol{W}}$ prompts the learned structural relationship as close to the original adjacency as possible. It is calculated as follows:


(6) \begin{align*} L_{bce} &= BCE\big(\widetilde{\boldsymbol{A}}, \overline{\boldsymbol{W}}\big) \nonumber \\ &= -\frac{1}{N^{2}} \sum_{i=1}^{N} \sum_{j=1}^{N} \left[ \widetilde{\boldsymbol{A}}_{ij} \cdot \log\big(\overline{\boldsymbol{W}}_{ij}\big) \right. \nonumber \\ &\quad \left. + (1-\widetilde{\boldsymbol{A}}_{ij}) \cdot \log\big(1-\overline{\boldsymbol{W}}_{ij}\big) \right]\end{align*}

Simultaneously, minimizing the Kullback–Leibler (KL) divergence between the learned local structural distribution and the prior distribution to enable the spots representation to contain more fine-grained semantic information, as follows:


(7) \begin{align*}& L_{k l}=K L(\overline{\boldsymbol{W}} \| \widetilde{\boldsymbol{A}})=\frac{1}{N} \sum_{i}^{N} \sum_{j}^{N} \overline{\boldsymbol{W}}_{i j} \log \frac{\overline{\boldsymbol{W}}_{i j}}{\widetilde{\boldsymbol{A}}_{i j}}\end{align*}

The overall loss $L_{G}$ of mutual information constraints of graph structures is formulated as:


(8) \begin{align*}& L_{G}=L_{bce}+L_{kl}\end{align*}

Moreover, to make the spots representation $\boldsymbol{Z}$ more informative and discriminative, an InfoNCE-like contrastive learning loss is calculated to bring spatially adjacent spots closer to each other and push spatially non-adjacent spots further apart in the latent space, as follows:


(9) \begin{align*}& L_{\textrm{con}}=-\frac{1}{N} \sum_{i}^{N} \log \frac{\sum_{j \in N_{i}}^{N} \exp^{\left(\cos \left(\boldsymbol{Z}_{i}, \boldsymbol{Z}_{j}\right) / \tau\right)}}{\sum_{j \notin N_{i}}^{N} \exp^{\left(\cos \left(\boldsymbol{Z}_{i}, \boldsymbol{Z}_{j}\right) / \tau\right)}}\end{align*}

where $N_{i}$ is the set of neighbors of spot $i$, $\cos \left (\boldsymbol{Z}_{i}, \boldsymbol{Z}_{j}\right )$ represents the cosine similarity between spot $i$ and $j$ in the latent embedding space and $\tau $ represents temperature parameter (set as 1 by default). For a given spot $i$, its neighbors are defined as positive pairs, while its non-neighbors are negative pairs.

### Model training strategy

In the process of learning spots representation, SpaGIC aims to minimize the overall loss, as follows:


(10) \begin{align*}& L=\lambda_{1}L_{mse}+\lambda_{2}L_{G}+\lambda_{3}L_{con}\end{align*}

where $\lambda _{1}$, $\lambda _{2}$,and $\lambda _{3}$ are weight factors that trade off each loss term. Their optimal values are determined by grid search (for details, see the section “The parameter settings of SpaGIC” in the Supplementary Notes.). Based on the results of the parameter analysis on the DLPFC dataset (Supplementary Fig. S1), the default values are set to 60, 0.01, and 0.01, respectively. The Adam optimizer [34] with a learning rate of 0.001 and a weight decay of 0.0001 is employed to optimize the model.

### Joint analysis of multiple slices with batch effect correction

With the development of ST technologies, the importance of performing joint analysis on multiple slices has increased significantly. In this context, SpaGIC can do this in combination with Harmony [28], a batch-effect correction method tailored for scRNA-seq data. Specifically, in vertical joint analysis, we construct the adjacency matrix $\widetilde{\boldsymbol{A}}^{k} \left (k=\left \{1,2,\ldots ,K\right \}\right )$ for each slice separately, with $\boldsymbol{X}^{k}$ denoting the gene expression matrix of the respective slice. Subsequently, all adjacency matrices $\widetilde{\boldsymbol{A}}^{k}$ are diagonally concatenated, while the gene expression matrices are concatenated in the spot dimensions, as follows:


(11) \begin{align*}& \widetilde{\boldsymbol{A}}=\left[\begin{array}{ccc} \widetilde{\boldsymbol{A}}^{1} & \cdots & 0 \\ \vdots & \ddots & \vdots \\ 0 & \cdots & \widetilde{\boldsymbol{A}}^{K} \end{array}\right] \quad X=\left[\begin{array}{c} \boldsymbol{X}^{1} \\ \vdots \\ \boldsymbol{X}^{K} \end{array}\right]\end{align*}

where $K$ is the number of slices. After obtaining the overall adjacency matrix $\widetilde{\boldsymbol{A}}$ and gene expression matrix $\boldsymbol{X}$, SpaGIC learns the spots representation $\boldsymbol{Z}$ following the pipeline of individual slice analysis.

To better correct for batch effects, we employ the *harmonypy* package for further processing to obtain batch-corrected spot embeddings $\boldsymbol{Z}^{hm}$. Then, $\boldsymbol{Z}^{hm}$ is used for multi-slice ST analysis, such as spatial clustering.

### Clustering and evaluation

In the workflow of SpaGIC, a variety of spatial domain identification strategies are provided based on the spots representation learned by SpaGIC. The clustering algorithms employed include mclust [35], k-means, Louvain [18], and Leiden. By default, the mclust algorithm is used by SpaGIC to cluster spots into different spatial domains. When ST datasets include the ground truth domain label, the number of spatial domains are set to be consistent with the number of real domain labels. However, in cases where the ground truth domain label is not available, the number of spatial domains is selected empirically.

After clustering, a refinement strategy is applied for some ST datasets, such as DLPFC and 10x Visium HBC, to achieve smoother domain boundaries. In this strategy, the domain label of a given spot is reset based on the label that is most frequently represented among its top-50 nearest neighbors in terms of spatial distance. It should be noted that this refinement strategy does not apply to all ST datasets.

The identified spatial domains are visualized with UMAP (Uniform Manifold Approximation and Projection) [36]. For datasets with manual annotations, the ARI (Adjusted Rank Index) [37] and NMI (Normalized Mutual Information) [38] are used to evaluate spatial clustering accuracy.

## Results

### SpaGIC effectively identifies spatial domains on 10x Visium data

**Figure 2:**
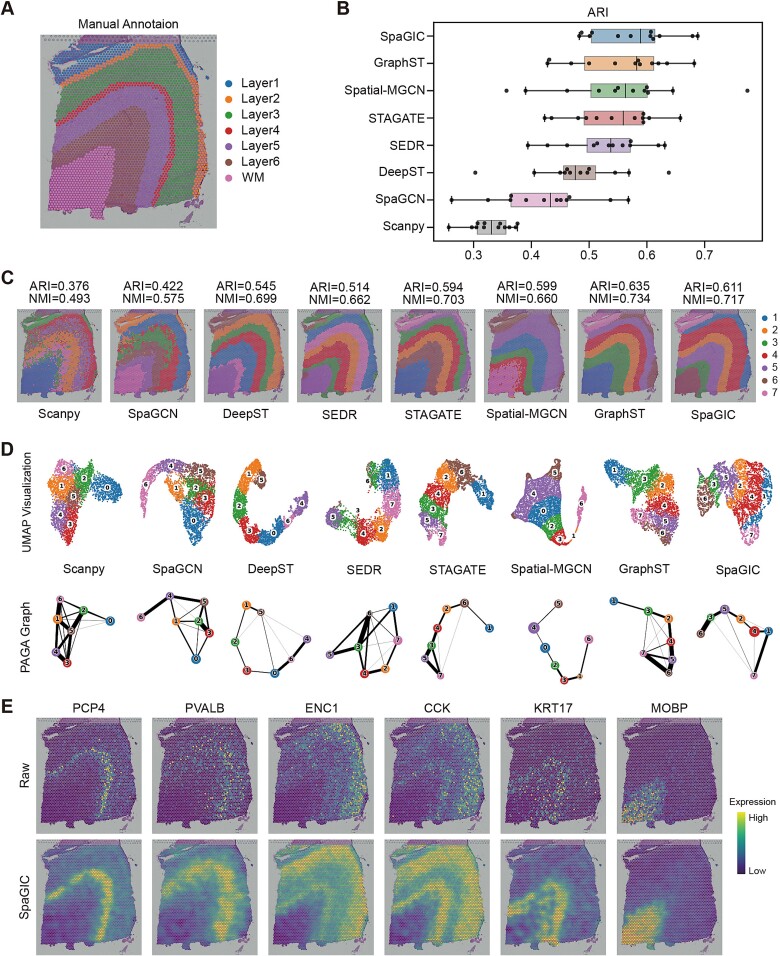
Spatial domains identification and data denoising on the DLPFC dataset. (**A**) Manual annotation of the DLPFC 151673 slice. (**B**) ARI boxplots of eight methods on 12 DLPFC slices. In the boxplot, the center line denotes the median, box limits denote the upper and lower quartiles, and whiskers denote the 1.5$\times $ interquartile range. (**C**) The spatial domains identified by Scanpy, SpaGCN, DeepST, SEDR, STAGATE, Spatial-MGCN, GraphST, and SpaGIC on the DLPFC 151673 slice. (**D**) UMAP visualization and PAGA graph generated based on the embedding by these methods on the 151673 slice. (**E**) Visualization of the raw expression of layer marker genes in the 151673 slice, both before and after denoising by SpaGIC.

SpaGIC was compared with existing state-of-the-art methods on multiple 10x Visium datasets, including DLPFC, HBC and mouse brain anterior (MBA), to demonstrate its effectiveness in identifying spatial domains. The baseline methods analyzed in this comparison are the Louvain algorithm implemented by Scanpy package and six other spatial domain identification algorithms (GraphST, DeepST, STAGATE, SpaGCN, SEDR and Spatial-MGCN). The overall experimental results showed that SpaGIC outperformed the baseline methods in spatial domain identification performance (Fig. 2 and Supplementary Fig. S2).

The DLPFC dataset was manually annotated by Maynard et al. [29] as the cortical layers (L1–L6) and WM. Taking it as the ground truth, we evaluated the clustering accuracy of SpaGIC and competing methods on 12 slices in terms of ARI and NMI. As shown in Fig. 2B, SpaGIC achieved the best performance across all 12 slices, with a median ARI of 0.589. Followed by GraphST and Spatial-MGCN, had median ARI of 0.563 and 0.56, respectively. The median ARI of the remaining methods were all less than 0.52, including DeepST, STAGATE, SpaGCN, and SEDR. Notably, SpaGIC also obtained the largest mean ARI of 0.576 and a smaller variance in ARI of 0.72 (Fig. 2B and Supplementary Table S2). This suggests that SpaGIC can detect both consistent tissue structures across different slices and slice-specific heterogeneity. Although Scanpy had the smallest variance, its overall ARI score was comparatively lower. Similar findings are observed for NMI (Supplementary Fig. S2A).

The spatial domain label refining strategy makes the domain boundary identified by SpaGIC clearer and smoother. For example, on the classic DLPFC 151673 slice, GraphST and SEDR, using a similar refinement strategy, showed similar results (Fig. 2C). The visual comparison clearly showed that SpaGIC, GraphST, and STAGATE all effectively identified the expected layered distribution of cortical layers, while the spatial domains identified by SpaGIC were more consistent with the true distribution of organizational structure (Fig. 2A). Although GraphST had slightly higher ARI and NMI scores on this slice, SpaGIC outperformed GraphST in ARI and NMI scores on 7 out of the 12 slices (Supplementary Table S2). In addition, Spatial-MGCN achieved high ARI scores, identified highly cluttered clusters in the WM and layer 6. SEDR detected most domains, but there existed a small cluster that did not have a clear correspondence with the cortical layers distribution. Cortical layers identified by DeepST were obviously misaligned with the ground truth. On the other hand, SpaGCN and Scanpy had the worst spatial clustering performance, only recovering layer 1 and the WM. The results of spatial domain identification for other slices can be seen in Supplementary Fig. S7.

The integration of edge-wise and local neighborhood-wise spatial information enables SpaGIC to better capture the spatial adjacency information of spots. The Fig. 2D showcased the results of spots embedding UMAP visualization and PAGA trajectory inference for SpaGIC and baseline methods on the DLPFC 151673 slice. Except Spatial-MGCN and DeepST, the UMAP plots and PAGA graph generated by embeddings of other baseline methods exhibited unclear hierarchical relationships and inconsistent spatial trajectories across cortical layers. In particular, the UMAP plots of Scanpy displayed very fuzzy cluster boundaries. By contrast, SpaGIC’s embedding UMAP plots and PAGA graph revealed spatial trajectories consistent with the functional similarity between adjacent cortical layers, following a chronological order from layer 1 to layer 6 and WM. The UMAP visualization and PAGA trajectory inference outcomes for other slices are detailed in Supplementary Fig. S8.

Moreover, the performance of SpaGIC in spatial clustering was further evaluated using the HBC dataset, which exhibits a more intricate spatial tissue structure. This dataset comprises four primary morphotypes: ductal carcinoma in situ/lobular carcinoma in situ (DCIS/LCIS), healthy tissue (Healthy), invasive ductal carcinoma (IDC), and tumor surrounding regions (Tumor edge). The results indicated a high level of consistency between the spatial domains identified by SpaGIC and manual annotation, such as clusters 2 (Tumor edge_1), 5 (Healthy_1), 7 (DCIS/LCIS_4), 9 (IDC_8), and 17 (IDC_4) (Supplementary Fig. S2D). Compared with domains identified by competing methods, SpaGIC detected domains with smoother boundaries and reduced noise. Additionally, in the analysis of the MBA dataset, SpaGIC detected the tissue structure closer to manual annotation, with higher ARI (0.443) and NMI (0.556) scores than the baseline methods (supplementary Fig. S2E). Overall, SpaGIC proves to be an effective tool for identifying spatial domains in 10x Visium datasets across diverse tissue types.

The gene expression profile of ST data is subject to high noise and dropout due to inherent technological limitations. By utilizing a GCN-based decoder, SpaGIC effectively reconstructs denoised and imputed gene expression profiles, enhancing the capture of spatial representation patterns. To validate its efficacy, SpaGIC was applied to the DLPFC 151673 slice, analyzing the expression distribution of six known layer-marker genes (PCP4, PVALB, ENC1, CCK, KRT17, MOBP) [29] in both the raw data and the expression matrix reconstructed by SpaGIC. As shown in Fig. 2E, these genes exhibited more distinct layer-specific expressions, consistent with previously reported results [39]. For example, after denoising, genes such as PCP4 and MOBP showed significantly different expressions in layer 4 and WM, respectively, compared to the raw data. Besides, visualization through violin plots further illustrated the differences between raw and imputed expressions by SpaGIC (Supplementary Fig. S2H–I). These plots revealed a heightened layer specificity and improved alignment with the real tissue structure in the denoised expression profiles. Collectively, these findings indicate that SpaGIC is a robust method that can mitigate noise influences and accurately impute gene expression data.

### SpaGIC better distinguishes the laminar structure of mouse olfactory bulb data from Stereo-seq and Slide-seqV2

**Figure 3:**
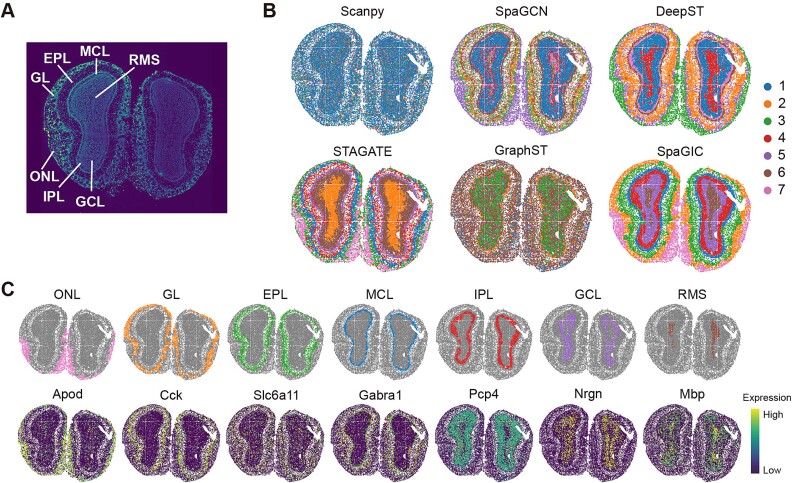
Spatial domains identification on the Stereo-seq MOB dataset. (**A**) The laminar structure of the Stereo-seq MOB annotated in DAPI-stained images. (**B**) Clustering results from Scanpy, SpaGCN, DeepST, STAGATE, GraphST, and SpaGIC. (**C**) Visualization of spatial domains detected by SpaGIC and related marker gene expression.

To verify the scalability of SpaGIC, we further evaluated the spatial clustering performance of SpaGIC on datasets obtained from platforms other than the 10x Visium platform. SpaGIC was applied to the MOB tissue with a laminar structure, including data profiled by Stereo-seq and Slide-seqV2, respectively. These platforms can capture gene expressions at a higher resolution, compared to the 10x Visium platform. The laminar structure of MOB Stereo-seq data was annotated based on previous research [15, 25] using a DAPI-stained image, including the rostral migratory stream (RMS), granule cell layer (GCL), internal plexiform layer (IPL), mitral cell layer (MCL), external plexiform layer (EPL), glomerular layer (GL), and olfactory nerve layer (ONL) (Fig. 3A). Scanpy clustering, shown in Fig. 3B, resulted in most spots being grouped into a single cluster, indicating the worst outcomes due to the lack of spatial information integration. SpaGCN only detected the ONL domain, with other domains being noisy. STAGTE distinguished the MCL and IPL domains but mixes the GCL and RMS regions. GraphST with default parameters showed inferior results compared to the original author’s findings, with more mixing between clusters. Both DeepST and SpaGIC successfully identified the complete laminar structure, but SpaGIC detected domains with clearer boundaries and less noise.

In addition, the results of SpaGIC were validated by the expression of some known marker. As shown in Fig. 3C, the clusters identified by SpaGIC were highly consistent with regions exhibiting high expression of known marker genes. This alignment further supported the reliability of SpaGIC’s results. The same evaluation on the MOB Slide-seqV2 data showed similar results (Supplementary Fig. S3). The spatial domains identified by SpaGIC aligned well with the Allen Reference Atlas [40] annotations, with only STAGATE and SpaGIC able to distinguish between AOB and AOBgr domains. Notably, the corresponding marker genes Nrgn and Fxy1d6 were highly expressed on the detected AOB and AOBgr domains, respectively, providing further support for the effectiveness of SpaGIC (Supplementary Fig. S3C). Overall, SpaGIC accurately identified the laminar structure of the MOB, aligning closely with manual annotation.

### SpaGIC finely detects the tissue structure of mouse visual cortex STARmap data and mouse somatosensory cortex osmFISH data

**Figure 4:**
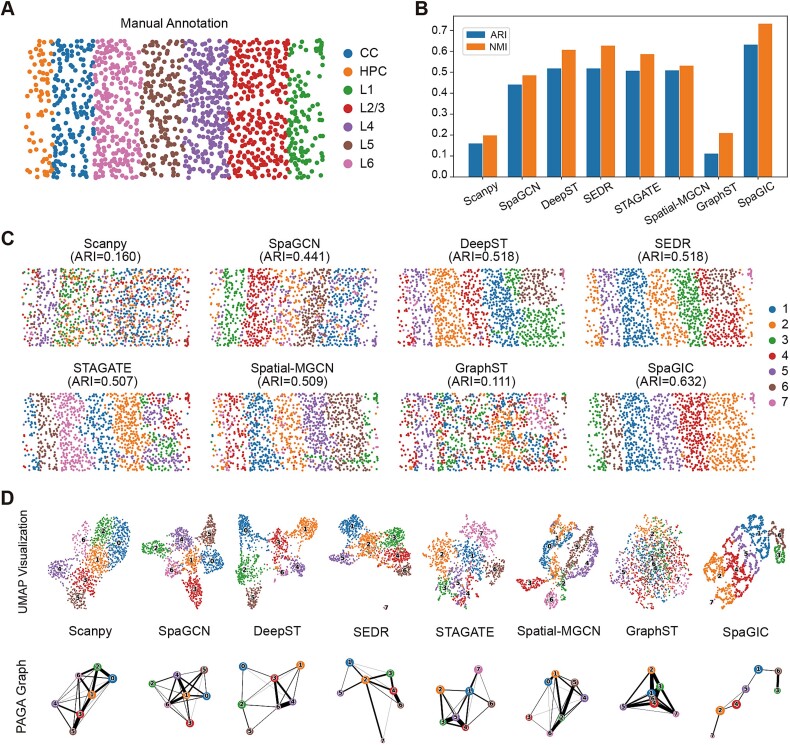
Spatial domains identification on the STARmap MVC dataset. (**A**) Manual annotation of the STARmap MVC. (**B**) ARI and NMI bar charts for eight methods. (**C**) The spatial domains identified by Scanpy, SpaGCN, DeepST, SEDR, STAGATE, Spatial-MGCN, GraphST, and SpaGIC. (**D**) UMAP visualization and PAGA graph generated by the embedding of these methods.

Following that, we further tested the performance of SpaGIC on datasets with low gene expression capture rates. One such dataset is the MVC dataset generated by STARmap, capturing the expression profiles of only 1020 genes across 1207 cells. The tissue structure and cell type distribution were provided by the original study (Fig. 4A). The experimental results revealed that spatial domains identified by SpaGIC aligned most accurately with the manually annotated layer structure, achieving the highest ARI score of 0.631 and NMI score of 0.732 (Fig. 4B-C). In contrast, baseline methods detected more cluttered spatial domains, with lower ARI scores (0.16 for Scanpy, 0.441 for SpaGCN, 0.518 for DeepST, 0.518 for SEDR, 0.507 for STAGATE, 0.509 for Spatial-MGCN, and 0.111 for GraphST) (Fig. 4C). Moreover, UMAP visualization and PAGA trajectory inference were performed based on the embedding of all methods. As shown in Fig. 4D, SpaGIC effectively separated all layers, with its PAGA graph exhibiting the highest consistency with the developmental orders among all methods.

SpaGIC was also employed to the MSC osmFISH dataset, which was manually annotated as containing 12 domains with 3405 cells and 33 genes. Based on previous study [41], our experiments focus on the cortical region, including ‘Layer 2–3 lateral’, ‘Layer 2–3 medium’, ‘Layer 3-4’, ‘Layer4’, ‘Layer5’, ‘Layer6’, and ‘Pia Layer1’ (Supplementary Fig. S4A). It is observed that SpaGIC identified spatial domains that are more consistent with manual annotations than baseline methods (Supplementary Fig. S4C). Particularly, SpaGIC clearly identified Pia Layer1, Layer5, and Layer6 with smoother boundaries and reduced noise. Although DeepST accurately detected Layer4, mixed the remaining layers together. Moreover, compared with other methods, SpaGIC achieved the highest ARI and NMI scores of 0.613 and 0.69, respectively, while the ARI scores of baseline methods were all below 0.52 (Supplementary Fig. S4B). These results demonstrate that SpaGIC can make full use of gene expression data for effective spatial clustering, even when the number of captured genes is limited.

Notably, we also analyzed the spatial domain identification performance of scTPC, a clustering method specifically designed for scRNA-seq data, on the MVC and MSC datasets. As expected, scTPC achieved sub-optimal performance because it did not incorporate spatial location information. However, it is undeniable that its ARI score on the MSC dataset surpassed that of some spatial clustering methods (Supplementary Fig. S5).

### SpaGIC performs multi-slice joint analysis combining Harmony while correcting batch effects

**Figure 5:**
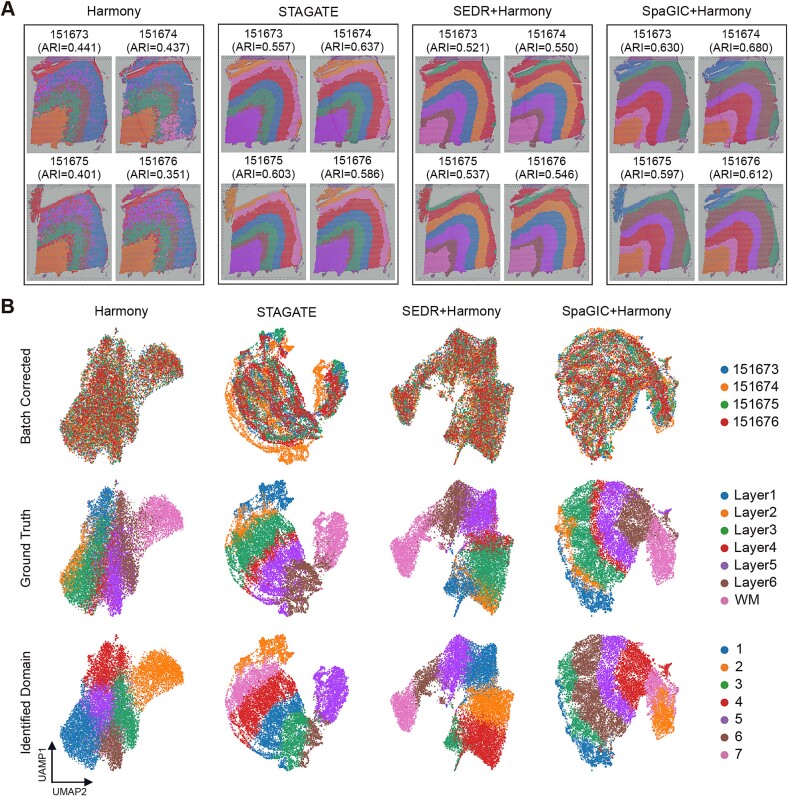
Joint analysis on the DLPFC dataset. (**A**) Aligned spatial domain identified by Harmony, STAGATE, SEDR, and SpaGIC via joint analysis of four slices of sample 3 (151673-151676). (**B**) UMAP visualization of embeddings colored by slices (top), ground truth (middle), and identified domains (bottom).

Integrated multi-slice analysis is one of the important studies in ST research, offering more in-depth insights. However, many existing methods can only be applied to the analysis of a single ST data slice. To evaluate the performance of SpaGIC in integration analysis, we applied it to the DLPFC dataset obtained from 10x Visium, which contains 12 slices from 3 samples, with each sample including 4 adjacent slices. Initially, SpaGIC was compared with STAGATE, SEDR, and Harmony using 4 adjacent slices from sample 3, including slices 151673, 151674, 151675, and 151676. In the UMAP visualization, all methods except STAGATE perfectly blended the 4 slices together (Fig. 5B). Compared with SEDR and Harmony, SpaGIC and STAGATE identified the common cortical layers across the slices with a more uniform mixing, resulting in clearer boundaries between layers that better align with the ground truth (Fig. 5B). In addition, the ARI score for each slice was calculated to quantitatively evaluate the integration performance of all methods. As shown in Fig. 5A, SpaGIC achieved the best performance, displaying cortical layer distributions that are highly consistent with manual annotations. While Harmony effectively corrected batch effects between slices, it struggled to identify clear cortical layers. More importantly, the integrated analysis results of SpaGIC surpassed those of single slice analysis, highlighting the capability of SpaGIC in effectively performing multi-slice joint analysis by leveraging common properties among multiple slices while preserving slice-specific heterogeneity.

The further evaluation of SpaGIC proceeded with the analysis of slices 151509, 151671, and 151675 from comparable locations in the three samples. Since the slices above exhibit a stronger batch effect, STAGATE was combined with Harmony for a comparative assessment. The results showed that, aside from STAGATE, the other methods effectively removed batch effects between the slices (Supplementary Fig. S6B). As shown in Supplementary Fig. S6A, Harmony suffered from serious noise and only separated the common WM region across the three slices. Notably, SpaGIC, STAGATE, and SEDR all identified layer structures that are well consistent with manual annotations. In terms of identifying layer boundaries and achieving superior clustering accuracy, SpaGIC outperformed STAGATE and SEDR, with an average ARI of 0.594. This once again proves that SpaGIC can be combined with Harmony for multi-slice joint analysis while correcting batch effects.

### Ablation study

To verify the effectiveness of the SpaGIC components, a series of ablation experiments were performed on the DLPFC dataset. Specifically, the edge-wise mutual information constraints, local neighborhood-wise mutual information constraints, and contrast learning constraints were respectively removed to evaluate the impact of each part on the overall performance of SpaGIC.

- SpaGIC-w/o-$L_{bce}$: it removes the global mutual information constraints, i.e. the overall loss $L$ does not include $L_{bce}$.
- SpaGIC-w/o-$L_{kl}$: it removes the local mutual information constraints, i.e. the overall loss $L$ does not include $L_{kl}$.
- SpaGIC-w/o-$L_{con}$: it removes the contrast learning constraints, i.e. the overall loss $L$ does not include $L_{con}$.
- SpaGIC-w/o-$L_{G}$: it removes the graph-based mutual information constraints, i.e. the overall loss $L$ does not include $L_{bce}$ and $L_{kl}$.
- SpaGIC-only-$L_{mse}$: it removes the constraints of self-supervised contrast learning and uses only the reconstruction loss.

**Figure 6:**
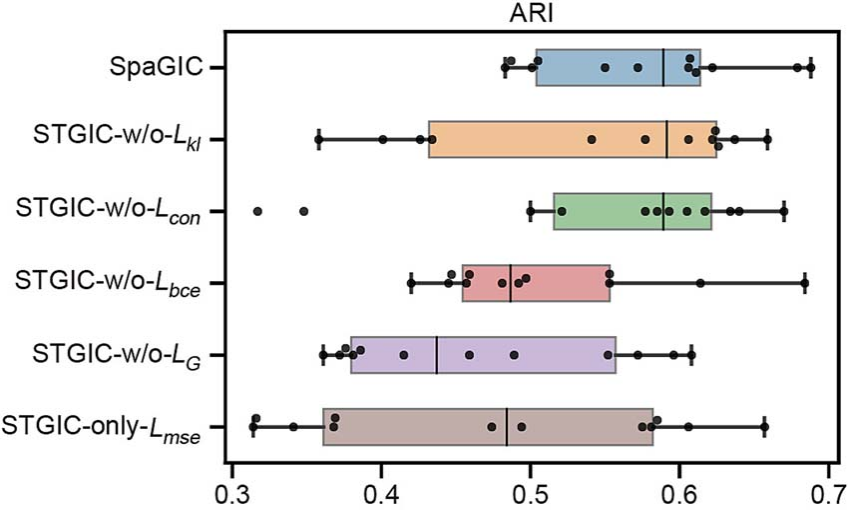
The ARI boxplots of SpaGIC and its variants on the DLPFC dataset.

As shown in Fig. 6, SpaGIC outperformed its variants in terms of ARI score. Although SpaGIC-w/o-$L_{kl}$ and SpaGIC-w/o-$L_{con}$ showcased higher median ARI scores (0.592 and 0.589, respectively) than that of SpaGIC (median ARI = 0.589, mean ARI = 0.576), their mean ARI values were lower (0.543 for SpaGIC-w/o-$L_{kl}$ and 0.551 for SpaGIC-w/o-$L_{con}$) with larger variance. Notably, the remaining two variants exhibited median ARI scores below 0.5. These results from ablation experiments demonstrated the significance of the individual components within SpaGIC. The combination of graph-based mutual information constraints and the contrast learning constraints synergistically empowers SpaGIC to effectively integrate spatial information with gene expression data, thereby enhancing its efficacy in ST analysis.

## Discussion and Conclusion

Effective identification of spatial domains forms the foundation for in-depth exploration of ST data. In this work, we propose SpaGIC, a self-supervised contrast learning framework for graph-informed clustering in ST. The framework models the ST data as a feature graph and uses a GCN-based auto-encoder to learn the latent representation of spots. To fully utilize the spatial information of ST data, SpaGIC constructs the adjacency relationship based on the spatial coordinate similarity between spots. The adjacency matrix is reconstructed using the latent representation of spots, and the information hidden in the graph structure is fully exploited by maximizing mutual information. SpaGIC considers both edge-wise mutual information and local neighborhood-wise mutual information. Furthermore, an InfoNCE-like loss is employed to promote the spots representation to be more discriminative and informative.

To validate the effectiveness of SpaGIC, comprehensive tests were performed on seven publicly available datasets from different platforms. The experimental results revealed that SpaGIC exhibited competitive performance when compared to existing state-of-the-art baseline methods. Notably, whether dealing with low-resolution ST data or high-resolution ST data with low gene capture rates, SpaGIC consistently and accurately identified spatial domains, demonstrating stable performance across different conditions. In MOBs experiments, it is observed that SpaGIC effectively detected the laminar structures. This success can be attributed to the fact that SpaGIC considers both edge-wise and local neighborhood-wise graph structural mutual information, which enables SpaGIC to incorporate more fine-grained information into the latent representation of spots. More importantly, SpaGIC successfully integrated with Harmony for efficient multi-slice joint analysis while effectively removing batch effects. Ablation experiments further confirmed the necessity of all components of SpaGIC, highlighting the importance of integrating all parts to construct a comprehensive and robust SpaGIC framework.

SpaGIC demonstrated excellent performance across several ST analysis tasks, such as spatial domain identification, data denoising, visualization, and trajectory inference. As ST technology continues to evolve and data scales increase, computational methods must meet the fundamental requirements of efficiency and scalability. We benchmarked the running time and GPU memory usage of SpaGIC using the Stereo-seq MOB dataset, and the results demonstrated that the time cost of SpaGIC is still acceptable (Supplementary Fig. S9A–C). However, SpaGIC is sensitive to GPU memory limitations, which somewhat limits its scalability for large datasets (Supplementary Fig. S9D). Future work is expected to enhance the scalability of the model by reconstructing partial non-adjacency relationships or by implementing techniques such as mini-batching and parallel computing. In addition, we will consider integrating ST and scRNA-seq data to broaden SpaGIC’s applications in cell type annotation tasks.

**Key Points**

- In this work, we propose SpaGIC, a graph-based self-supervised contrast learning framework for ST analysis, including spatial domain identification, data denoising, visualization, trajectory inference, and multi-slice joint analysis.
- SpaGIC constructs a feature graph that integrates spatial information and gene expression profiles. Using a GCN-based auto-encoder, SpaGIC learns the latent representation of spots. Meanwhile, graph structural mutual information constraints and an InfoNCE-like loss are introduced to make the spots representation more discriminative and informative.
- We evaluated SpaGIC on seven publicly available datasets from various platforms. The experimental results demonstrated that SpaGIC achieved competitive performance when compared to existing state-of-the-art baseline methods.

## Supplementary Material

SpaGIC_supplementary_material_bbae578

## Data Availability

All datasets used in this work can be downloaded from the link below. Specifically, (i) the LIBD human dorsolateral prefrontal cortex (DLPFC) dataset: http://spatial.libd.org/spatialLIBD/; (ii) the 10x Visium human breast cancer dataset: https://www.10xgenomics.com/datasets/human-breast-cancer-block-a-section-1-1-standard-1-1-0; (iii) the anterior section of the 10x Visium mouse brain: https://www.10xgenomics.com/resources/datasets/mouse-brain-serial-section-1-sagittal-anterior-1-standard-1-1-0; (iv) the Stereo-seq mouse olfactory bulb dataset: https://github.com/STOmics/SAW/tree/main/Test_Data; (v) the Slide-seqV2 mouse olfactory bulb dataset: https://singlecell.broadinstitute.org/single_cell/study/SCP815/highly-sensitive-spatial-transcriptomics-at-near-cellular-resolution-with-slide-seqv2#study-summary; (vi) the STARmap mouse visual cortex dataset: https://drive.google.com/drive/folders/1I1nxheWlc2RXSdiv24dex3YRaEh780my?usp=sharing; (vii) the osmFISH mouse somatosensory cortex dataset: https://linnarssonlab.org/osmFISH/.

## Code availability

The code of SpaGIC can be downloaded from https://github.com/liuwei-cs/SpaGIC.

## References

[1] Asp M , Bergenstråhle J, Lundeberg J. Spatially resolved transcriptomes—next generation tools for tissue exploration. *Bioessays* 2020;42:e1900221. 10.1002/bies.201900221. 32363691

[2] Armingol E , Officer A, Harismendy O. et al. Deciphering cell–cell interactions and communication from gene expression. *Nat Rev Genet* 2021;22:71–88. 10.1038/s41576-020-00292-x. 33168968 PMC7649713

[3] Crosetto N , Bienko M, Van Oudenaarden A. Spatially resolved transcriptomics and beyond. *Nat Rev Genet* 2015;16:57–66. 10.1038/nrg3832. 25446315

[4] Hunter MV , Moncada R, Weiss JM. et al. Spatially resolved transcriptomics reveals the architecture of the tumor-microenvironment interface. *Nat Commun* 2021;12:6278. 10.1038/s41467-021-26614-z. 34725363 PMC8560802

[5] Rodriques SG , Stickels RR, Goeva A. et al. Slide-seq: a scalable technology for measuring genome-wide expression at high spatial resolution. *Science* 2019;363:1463–7. 10.1126/science.aaw1219. 30923225 PMC6927209

[6] Stickels RR , Murray E, Kumar P. et al. Highly sensitive spatial transcriptomics at near-cellular resolution with Slide-seqV2. *Nat Biotechnol* 2021;39:313–9. 10.1038/s41587-020-0739-1. 33288904 PMC8606189

[7] Ji AL , Rubin AJ, Thrane K. et al. Multimodal analysis of composition and spatial architecture in human squamous cell carcinoma. *Cell* 2020;182:497–514.e22. 10.1016/j.cell.2020.05.039. 32579974 PMC7391009

[8] Chen A , Liao S, Cheng M. et al. Spatiotemporal transcriptomic atlas of mouse organogenesis using DNA nanoball-patterned arrays. *Cell* 2022;185:1777–1792.e21. 10.1016/j.cell.2022.04.003. 35512705

[9] Lubeck E , Coskun AF, Zhiyentayev T. et al. Single-cell in situ RNA profiling by sequential hybridization. *Nat Methods* 2014;11:360–1. 10.1038/nmeth.2892. 24681720 PMC4085791

[10] Shah S , Lubeck E, Zhou W. et al. In situ transcription profiling of single cells reveals spatial organization of cells in the mouse hippocampus. *Neuron* 2016;92:342–57. 10.1016/j.neuron.2016.10.001. 27764670 PMC5087994

[11] Moffitt JR , Bambah-Mukku D, Eichhorn SW. et al. Molecular, spatial, and functional single-cell profiling of the hypothalamic preoptic region. *Science* 2018;362:eaau5324. 10.1126/science.aau5324. 30385464 PMC6482113

[12] Chen KH , Boettiger AN, Moffitt JR. et al. Spatially resolved, highly multiplexed rna profiling in single cells. *Science* 2015;348:aaa6090. 10.1126/science.aaa6090. 25858977 PMC4662681

[13] Codeluppi S , Borm LE, Zeisel A. et al. Spatial organization of the somatosensory cortex revealed by osmfish. *Nat Methods* 2018;15:932–5. 10.1038/s41592-018-0175-z. 30377364

[14] Wang X , Allen WE, Wright MA. et al. Three-dimensional intact-tissue sequencing of single-cell transcriptional states. *Science* 2018;361:eaat5691. 10.1126/science.aat5691. 29930089 PMC6339868

[15] Dong K , Zhang S. Deciphering spatial domains from spatially resolved transcriptomics with an adaptive graph attention auto-encoder. *Nat Commun* 2022;13:1739. 10.1038/s41467-022-29439-6. 35365632 PMC8976049

[16] Cheng Y , Ma X. scGAC: a graph attentional architecture for clustering single-cell rna-seq data. *Bioinformatics* 2022;38:2187–93. 10.1093/bioinformatics/btac099. 35176138

[17] Qiu Y , Yang L, Jiang H. et al. scTPC: a novel semisupervised deep clustering model for scRNA-seq data. *Bioinformatics* 2024;40:btae293. 38684178 10.1093/bioinformatics/btae293 PMC11091743

[18] Blondel VD , Guillaume J-L, Lambiotte R. et al. Fast unfolding of communities in large networks. *J Stat Mech Theory Exp* 2008;2008:P10008. 10.1088/1742-5468/2008/10/P10008.

[19] Zhao E , Stone MR, Ren X. et al. Spatial transcriptomics at subspot resolution with bayesspace. *Nat Biotechnol* 2021;39:1375–84. 10.1038/s41587-021-00935-2. 34083791 PMC8763026

[20] Dries R , Zhu Q, Dong R. et al. Giotto: a toolbox for integrative analysis and visualization of spatial expression data. *Genome Biol* 2021;22:1–31. 33685491 10.1186/s13059-021-02286-2 PMC7938609

[21] Chang X , Jin X, Wei S. et al. Deepst: identifying spatial domains in spatial transcriptomics by deep learning. *Nucleic Acids Res* 2022;50:e131–1. 36250636 10.1093/nar/gkac901 PMC9825193

[22] Hang X , Huazhu F, Long Y. et al. Unsupervised spatially embedded deep representation of spatial transcriptomics. *Genome Med* 2024;16:12. 10.1186/s13073-024-01283-x. 38217035 PMC10790257

[23] Jian H , Li X, Coleman K. et al. SpaGCN: integrating gene expression, spatial location and histology to identify spatial domains and spatially variable genes by graph convolutional network. *Nat Methods* 2021;18:1342–51. 34711970 10.1038/s41592-021-01255-8

[24] Li J , Chen S, Pan X. et al. Cell clustering for spatial transcriptomics data with graph neural networks. *Nat Comput Sci* 2022;2:399–408. 10.1038/s43588-022-00266-5. 38177586

[25] Long Y , Ang KS, Li M. et al. Spatially informed clustering, integration, and deconvolution of spatial transcriptomics with GraphST. *Nat Commun* 2023;14:1155. 10.1038/s41467-023-36796-3. 36859400 PMC9977836

[26] Wang B , Luo J, Liu Y. et al. Spatial-MGCN: a novel multi-view graph convolutional network for identifying spatial domains with attention mechanism. *Brief Bioinform* 2023;24:bbad262. 37466210 10.1093/bib/bbad262

[27] Veli P , Fedus W, Hamilton WL. et al. Deep Graph Infomax. In International Conference on Learning Representations. New Orleans, Louisiana, USA, 2019. https://openreview.net/forum?id=rklz9iAcKQ.

[28] Korsunsky I , Millard N, Fan J. et al. Fast, sensitive and accurate integration of single-cell data with harmony. *Nat Methods* 2019;16:1289–96. 10.1038/s41592-019-0619-0. 31740819 PMC6884693

[29] Maynard KR , Collado-Torres L, Weber LM. et al. Transcriptome-scale spatial gene expression in the human dorsolateral prefrontal cortex. *Nat Neurosci* 2021;24:425–36. 10.1038/s41593-020-00787-0. 33558695 PMC8095368

[30] Buache E , Etique N, Alpy F. et al. Deficiency in trefoil factor 1 (TFF1) increases tumorigenicity of human breast cancer cells and mammary tumor development in TFF1-knockout mice. *Oncogene* 2011;30:3261–73. 10.1038/onc.2011.41. 21358676 PMC3141110

[31] Wolf FA , Angerer P, Theis FJ. Scanpy: large-scale single-cell gene expression data analysis. *Genome Biol* 2018;19:1–5. 29409532 10.1186/s13059-017-1382-0 PMC5802054

[32] Kipf TN , Welling M. Semi-supervised classification with graph convolutional networks. In International Conference on Learning Representations. Toulon, France, 2017. https://openreview.net/forum?id=SJU4ayYgl.

[33] Zhao W , Gongping X, Cui Z. et al. Deep graph structural infomax. In *Proceedings of the AAAI Conference on Artificial Intelligence*. Washington, D.C., USA: AAAI, 2023, Vol. 37, pp. 4920–8. 10.1609/aaai.v37i4.25618.

[34] Kingma DP , Ba J. Adam: a method for stochastic optimization. arXiv preprint arXiv:1412.6980. 2014. 10.48550/arXiv.1412.6980.

[35] Fraley C , Raftery AE, Brendan Murphy T. et al. Mclust Version 4 for R: Normal Mixture Modeling for Model-Based Clustering, Classification, and Density Estimation Technical report. Citeseer, 2012.

[36] Becht E , McInnes L, Healy J. et al. Dimensionality reduction for visualizing single-cell data using UMAP. *Nat Biotechnol* 2019;37:38–44. 10.1038/nbt.4314. 30531897

[37] Rand WM . Objective criteria for the evaluation of clustering methods. *J Am Stat Assoc* 1971;66:846–50. 10.1080/01621459.1971.10482356.

[38] Amelio A , Pizzuti C. Correction for closeness: adjusting normalized mutual information measure for clustering comparison. *Comput Intell* 2017;33:579–601. 10.1111/coin.12100.

[39] Zuo C , Zhang Y, Cao C. et al. Elucidating tumor heterogeneity from spatially resolved transcriptomics data by multi-view graph collaborative learning. *Nat Commun* 2022;13:5962. 10.1038/s41467-022-33619-9. 36216831 PMC9551038

[40] Sunkin SM , Ng L, Lau C. et al. Allen brain atlas: an integrated spatio-temporal portal for exploring the central nervous system. *Nucleic Acids Res* 2012;41:D996–1008. 10.1093/nar/gks1042. 23193282 PMC3531093

[41] Shi X , Zhu J, Long Y. et al. Identifying spatial domains of spatially resolved transcriptomics via multi-view graph convolutional networks. *Brief Bioinform* 2023;24:bbad278. 10.1093/bib/bbad278. 37544658

