# Supplementary material for: SpaGIC: graph-informed clustering in spatial transcriptomics via self-supervised contrastive learning
Source: Brief Bioinform. 2024 Nov 14;25(6):bbae578. doi: 10.1093/bib/bbae578 (PMC11562840; doi:10.1093/bib/bbae578)
Supplement: SpaGIC_supplementary_material_bbae578 [file spagic_supplementary_material_bbae578.pdf]

Supplementary Materials for

SpaGIC: Graph-informed clustering in spatial transcriptomics via self-supervised contrastive learning

Wei Liu, Bo Wang, Yuting Bai, Xiao Liang, Li Xue and Jiawei Luo*

College of Computer Science and Electronic Engineering, Hunan University, Changsha 410083, China

*Corresponding author. Email: luojiawei@hnu.edu.cn

Keywords: spatial transcriptomics, spatial domain identification, graph convolutional networks, self-supervised contrastive learning

Contents

Supplementary Notes 3

Comparison with baseline methods 3

The parameter settings of SpaGIC 4

Supplementary Figures 5

Figure S1. Parametric analysis on the DLPFC dataset. (**A**) Median ARI under different conditions. (**B**) Mean ARI under different conditions. (**C**) Median NMI under different conditions. (**D**) Mean NMI under different conditions. 5

Figure S2. (**A**) NMI boxplots of eight methods (Scanpy, SpaGCN, DeepST, SEDR, STAGATE, Spatial-MGCN, GraphST and SpaGIC) on 12 DLPFC slices. (**B-C**) ARI and NMI bar charts for eight methods on HBC and MBA datasets, respectively. (**D-E**) Manual annotations of HBC and MBA datasets, respectively. (**F-G**) Spatial domains identified by eight methods on HBC and MBA datasets, respectively. (**H-I**) The violin plot of the raw expressions of layer-marker genes in DLPFC 151673 slice and the imputed expressions by SpaGIC, respectively. 6

Figure S3. (**A**) Laminar structure of the Slide-seqV2 MOB annotated in the Allen Reference Atlas. (**B**) The clustering results of Scanpy, SpaGCN, DeepST, STAGATE, GraphST and SpaGIC. (**C**) Visualization of spatial domains detected by SpaGIC and related marker gene expression. 7

Figure S4. (**A**) Manual annotation of the osmFISH MSC. (**B**) ARI and NMI bar charts for eight methods (Scanpy, SpaGCN, DeepST, SEDR, STAGATE, Spatial-MGCN, GraphST and SpaGIC). (**C**) The spatial domains identified by the above methods. 8

Figure S5. The spatial domains identified by scTPC on the MVC and MSC datasets. (**A**) Clustering results on the MVC dataset. (**B**) UMAP visualization and PAGA graph based on the scTPC embedding of the MVC dataset. (**C**) Clustering results on the MSC dataset. 9

Figure S6. (**A**) Aligned spatial domain identified by Harmony, STAGATE, SEDR and SpaGIC via joint analysis of slices 151509, 151671 and 151675 taken from similar locations in the 3 samples. (**B**) UMAP visualization of embeddings colored by slices (left), ground truth (middle) and identified domains (right) 9

Figure S7. Manual annotations and comparison of spatial domains identified by Scanpy, SpaGCN, DeepST, SEDR, STAGATE, Spatial-MGCN, GraphST and SpaGIC on the 12 DLPFC slices. 10

Figure S8. UMAP visualization and PAGA graphs generated by Scanpy, SpaGCN, DeepST, SEDR, STAGATE, Spatial-MGCN, GraphST and SpaGIC embeddings respectively. 12

Figure S9. Running time and GPU memory usage on the randomly sampled datasets derived from the Stereo-seq MOB dataset, which includes different numbers of spots, based on a server equipped with an Intel(R) Xeon(R) Silver 4208 CPU @ 2.10GHz and an NVIDIA GeForce RTX 4090 GPU. (**A-C**) Preprocessing time, training time, total time, respectively. (**D**) GPU memory usage 13

Supplementary Tables 14

Table S1. Summary of all datasets used in this study. 14

Table S2. ARI and NMI scores of Scanpy, SpaGCN, DeepST, SEDR, STAGATE, Spatial-MGCN, GraphST and SpaGIC on the DLPFC dataset. 15

Supplementary Notes

Comparison with baseline methods

To evaluate the performance of SpaGIC in spatial clustering, we compared SpaGIC with Louvain algorithm implemented by Scanpy package and six spatial clustering algorithms (GraphST, DeepST, STAGATE, SpaGCN, SEDR and Spatial-MGCN). To showcase the effectiveness of SpaGIC in multi-slice joint analysis, we compared SpaGIC with Harmony, STAGATE and SEDR. All baselines above run with the original default parameters and use the same number of clusters during clustering.

- **Scanpy**: The data preprocessing for SCANPY is the same as for SpaGIC. Then *scanpy.tl.pca()* was used for dimensionality reduction to obtain the first 50 principal components (PCs). Next, the nearest neighbor network was constructed using the *scanpy.pp.neighbor()* function with default parameters. Finally, SCANPY obtains the clustering results using the *scanpy.tl.louvain()* function.
- **GraphST**: GraphST is used for comparative analysis as described in their online tutorials. https://deepst-tutorials.readthedocs.io/en/latest/index.html
- **DeepST**: DeepST is used for comparative analysis as described in their online tutorials. https://github.com/JiangBioLab/DeepST

  It should be noted that the results of DeepST are unstable, so we chose a random set of results to compare.
- **STAGATE**: STAGATE is used for comparative analysis as described in their online tutorials. https://stagate.readthedocs.io/en/latest/index.html
- **SpaGCN**: SpaGCN is used for comparative analysis as described in their online tutorials. https://github.com/jianhuupenn/SpaGCN/tree/master/tutorial
- **SEDR**: SEDR is used for comparative analysis as described in their online tutorials. https://github.com/JinmiaoChenLab/SEDR/
- **Spatial-MGCN**: Spatial-MGCN is used for comparative analysis as described in their online tutorials. https://github.com/cs-wangbo/Spatial-MGCN

  It should be noted that when Spatial-MGCN is applied to a dataset with manual

annotations, the returned result is the best one observed during the training process, and not necessarily the final result.

- **Harmony**: Harmony is a nonspatial batch correction method. We used *Harmonypy* for the joint analysis of DLPFC. The data were processed following the same preprocessing pipeline as SpaGIC, and then PCA dimension reduction was carried out. The PCA embeddings and sample batch information were used as inputs to *Harmonypy* to obtain the batch-corrected embeddings.

The parameter settings of SpaGIC

For different datasets, we fine-tune the some hyperparameters of SpaGIC, as follows.

Dataset	λ_1	λ_2	λ_3	epochs	n_neighbor
DLPFC	60	0.01	0.01	500	5
HBC	60	0.01	0.01	200	5
MBA	60	0.01	0.01	200	5
MOB_Stereo-seq	5	0.01	0.01	500	15
MOB_Slide-seqV2	5	0.01	0.01	200	10
MVC	60	0.1	1	200	10
MSC	60	0.01	0.01	200	14

Supplementary Figures

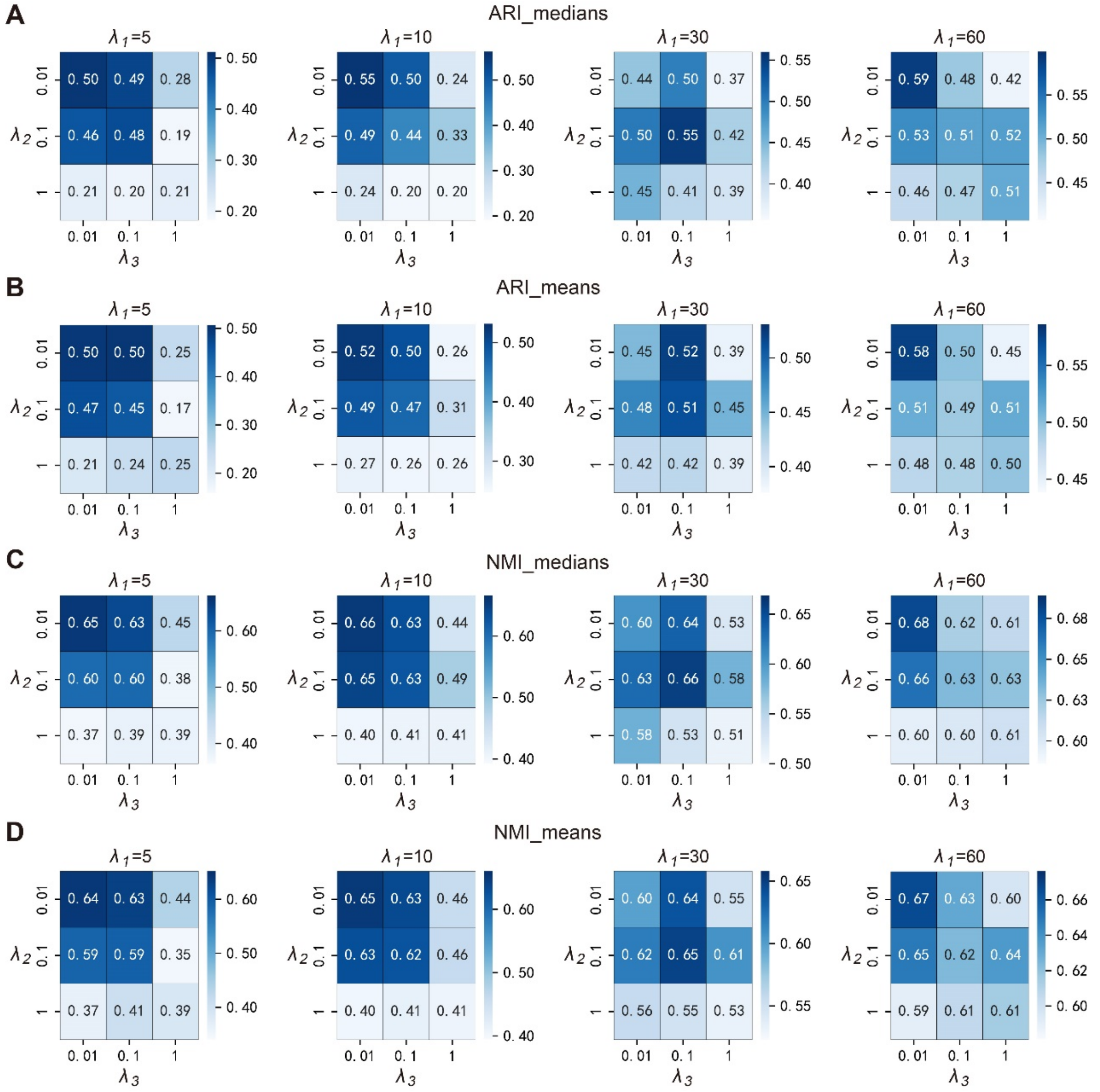

Figure S1. Parametric analysis on the DLPFC dataset. (**A**) Median ARI under different conditions. (**B**) Mean ARI under different conditions. (**C**) Median NMI under different conditions. (**D**) Mean NMI under different conditions.

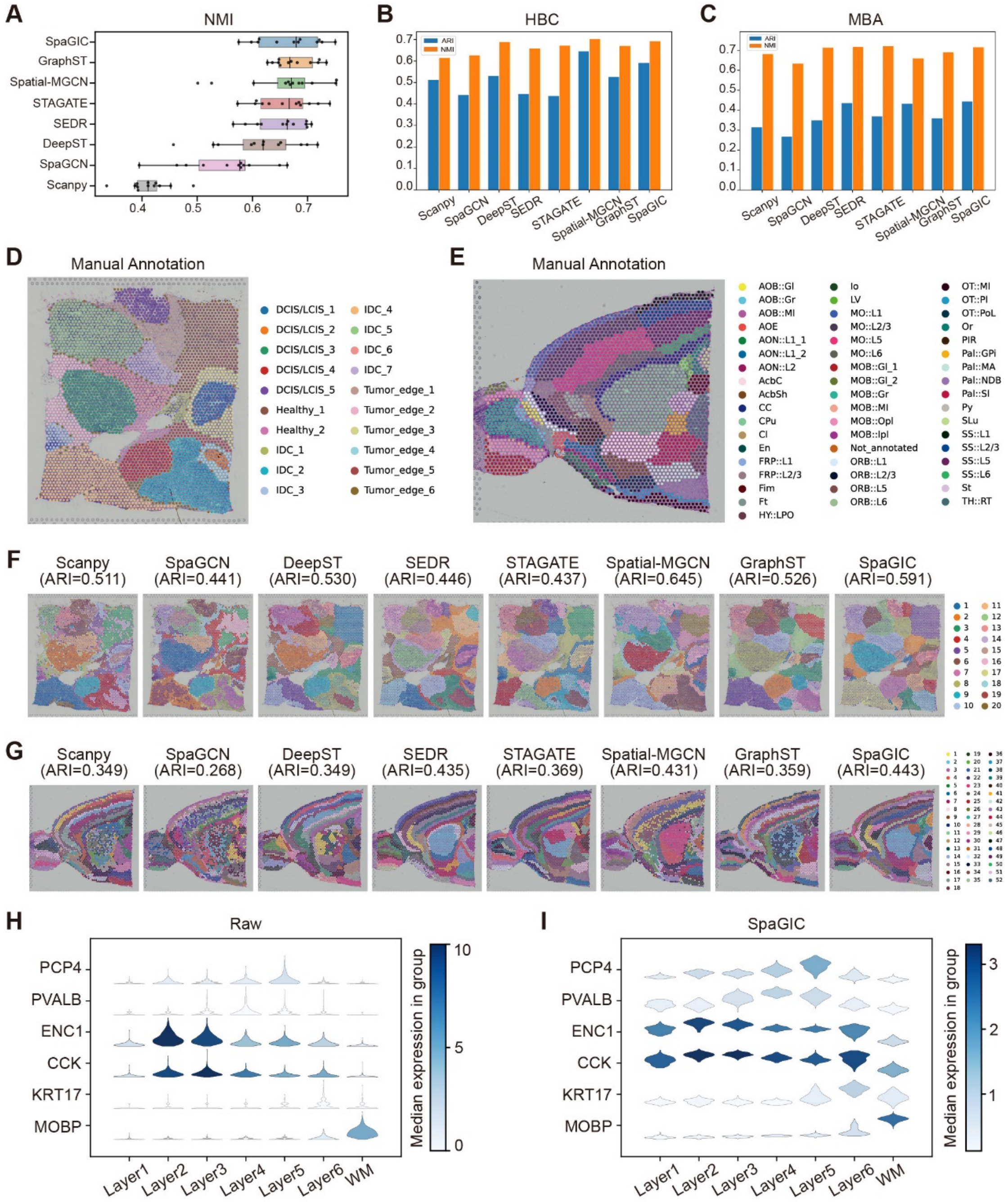

Figure S2. (**A**) NMI boxplots of eight methods (Scanpy, SpaGCN, DeepST, SEDR, STAGATE, Spatial-MGCN, GraphST and SpaGIC) on 12 DLPFC slices. (**B-C**) ARI and NMI bar charts for eight methods on HBC and MBA datasets, respectively. (**D-E**) Manual annotations of HBC and MBA datasets, respectively. (**F-G**) Spatial domains identified by eight methods on HBC and MBA datasets, respectively. (**H-I**) The violin plot of the raw expressions of layer-marker genes in DLPFC 151673 slice and the imputed expressions by SpaGIC, respectively.

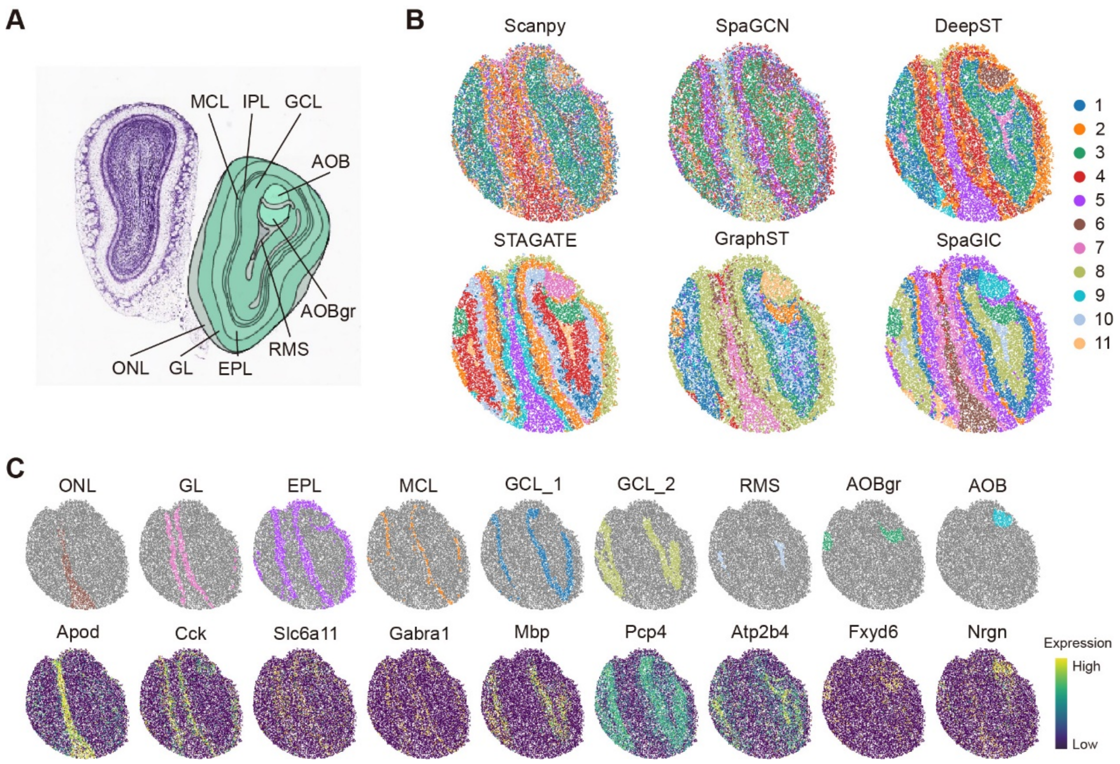

Figure S3. (**A**) Laminar structure of the Slide-seqV2 MOB annotated in the Allen Reference Atlas. (**B**) The clustering results of Scanpy, SpaGCN, DeepST, STAGATE, GraphST and SpaGIC. (**C**) Visualization of spatial domains detected by SpaGIC and related marker gene expression.

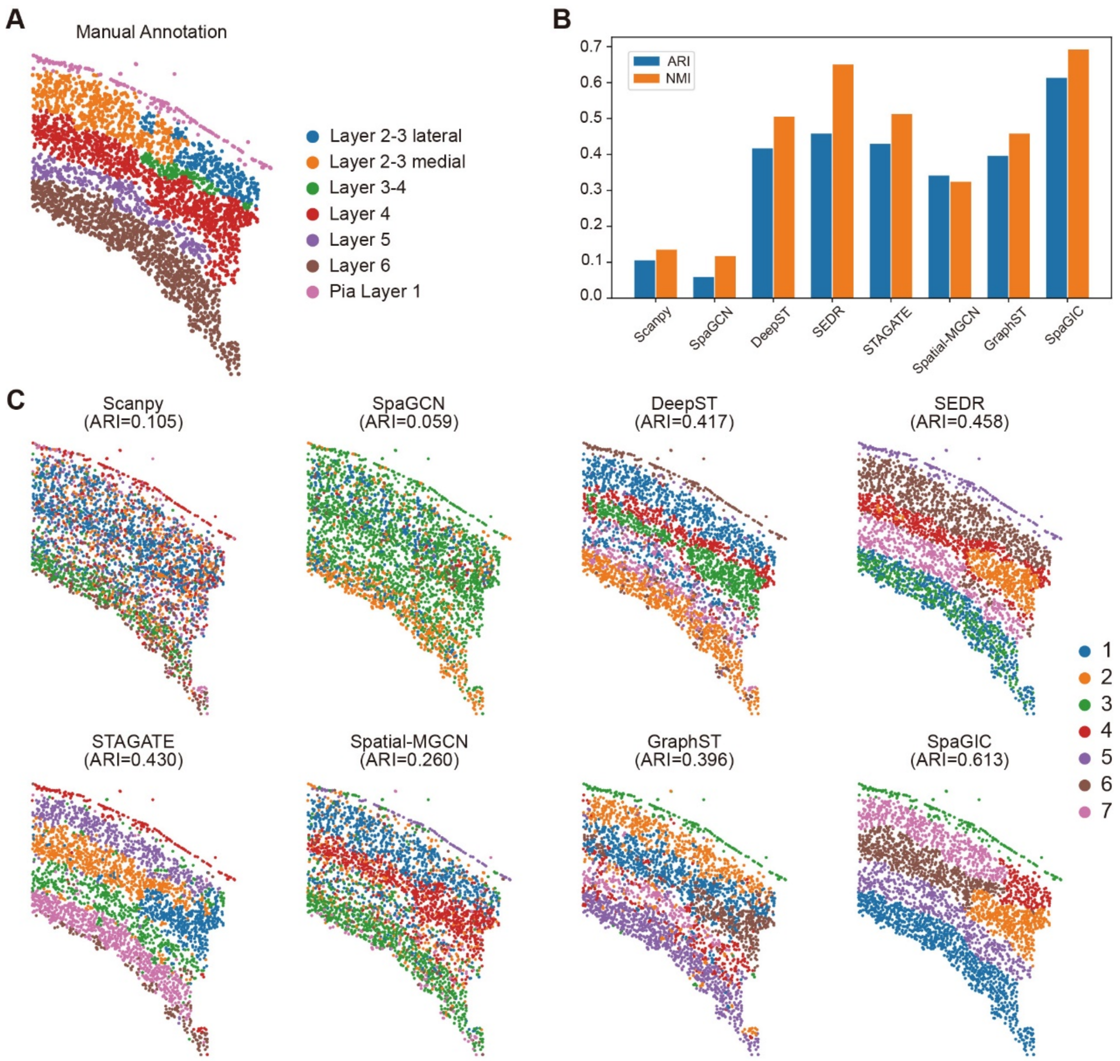

Figure S4. (**A**) Manual annotation of the osmFISH MSC. (**B**) ARI and NMI bar charts for eight methods (Scanpy, SpaGCN, DeepST, SEDR, STAGATE, Spatial-MGCN, GraphST and SpaGIC). (**C**) The spatial domains identified by the above methods.

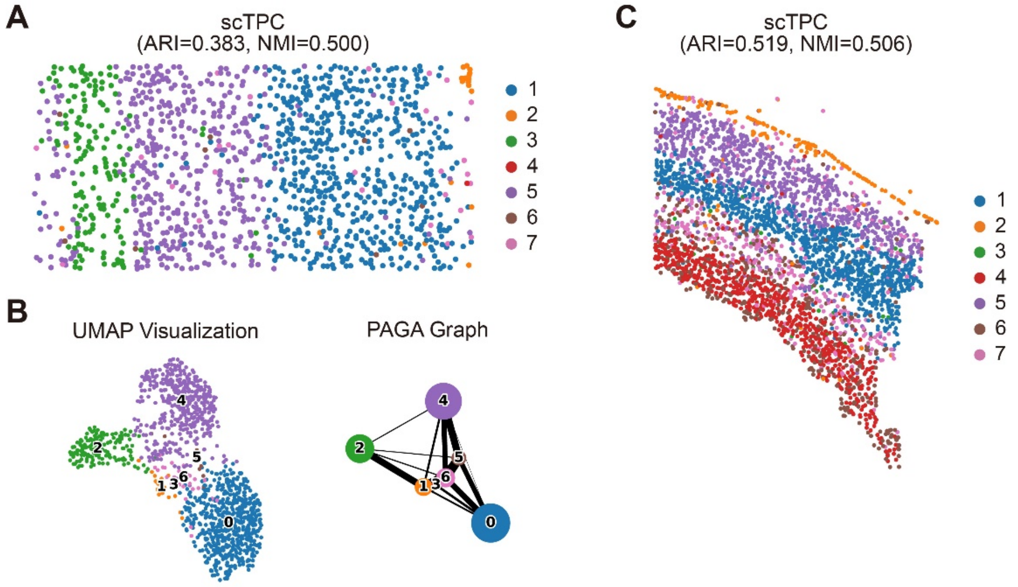

Figure S5. The spatial domains identified by scTPC on the MVC and MSC datasets. (**A**) Clustering results on the MVC dataset. (**B**) UMAP visualization and PAGA graph based on the scTPC embedding of the MVC dataset. (**C**) Clustering results on the MSC dataset.

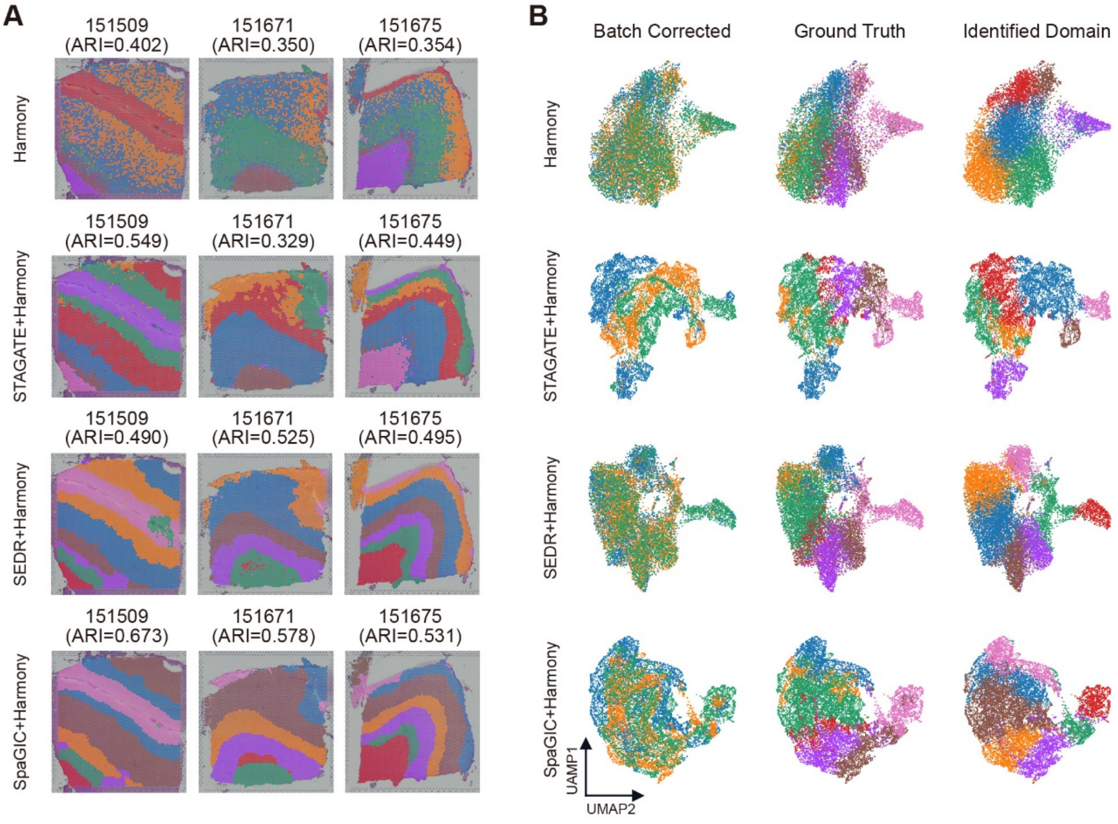

Figure S6. (**A**) Aligned spatial domain identified by Harmony, STAGATE, SEDR and SpaGIC via joint analysis of slices 151509, 151671 and 151675 taken from similar locations in the 3 samples. (**B**) UMAP visualization of embeddings colored by slices (left), ground truth (middle) and identified domains (right).

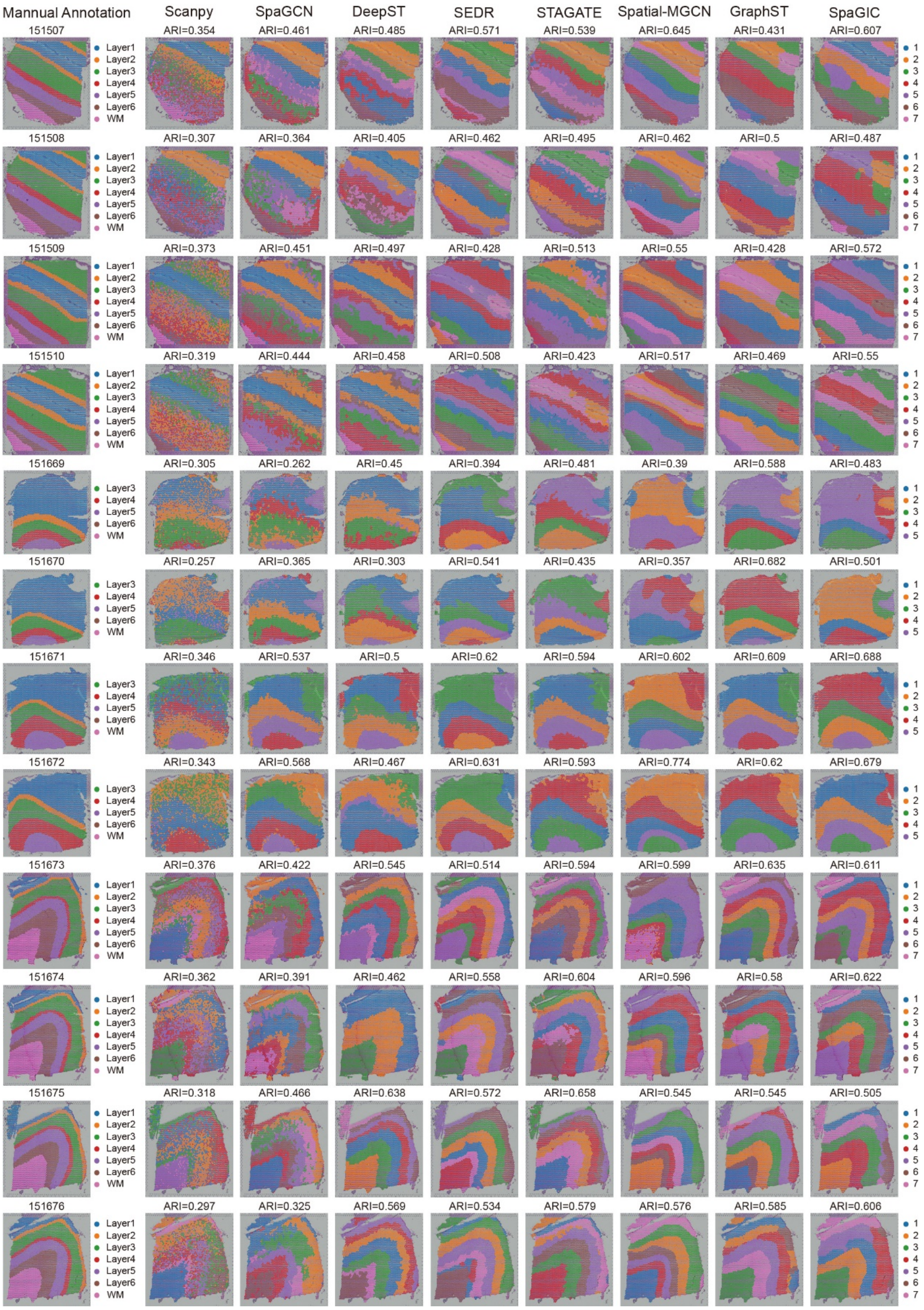

Figure S7. Manual annotations and comparison of spatial domains identified by Scanpy, SpaGCN, DeepST, SEDR, STAGATE, Spatial-MGCN, GraphST and SpaGIC on the 12 DLPFC slices.

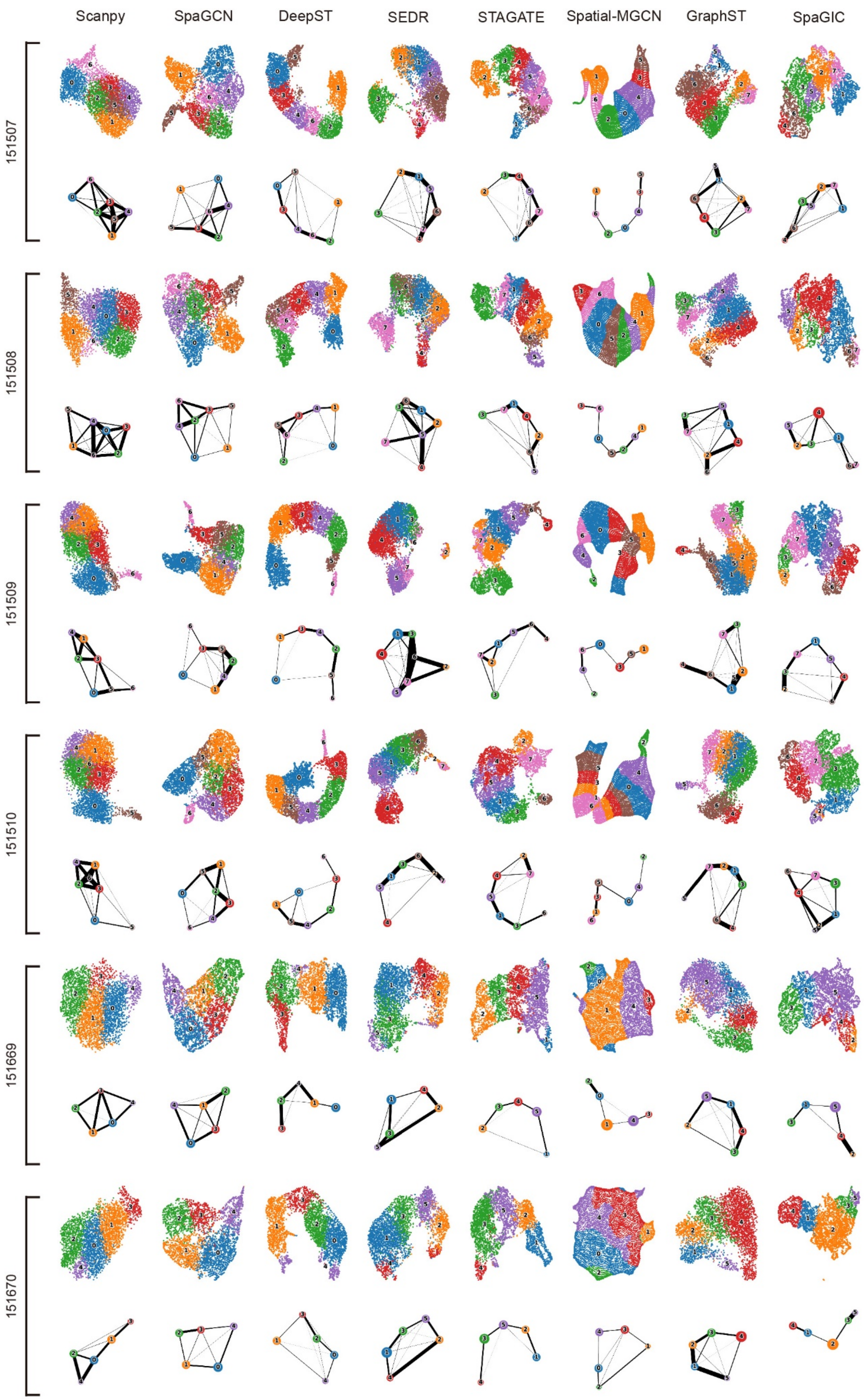
Scanpy
SpaGCN
DeepST
SEDR
STAGATE
Spatial-MGCN
GraphST
SpaGIC
151507
151508
151509
151510
151669
151670

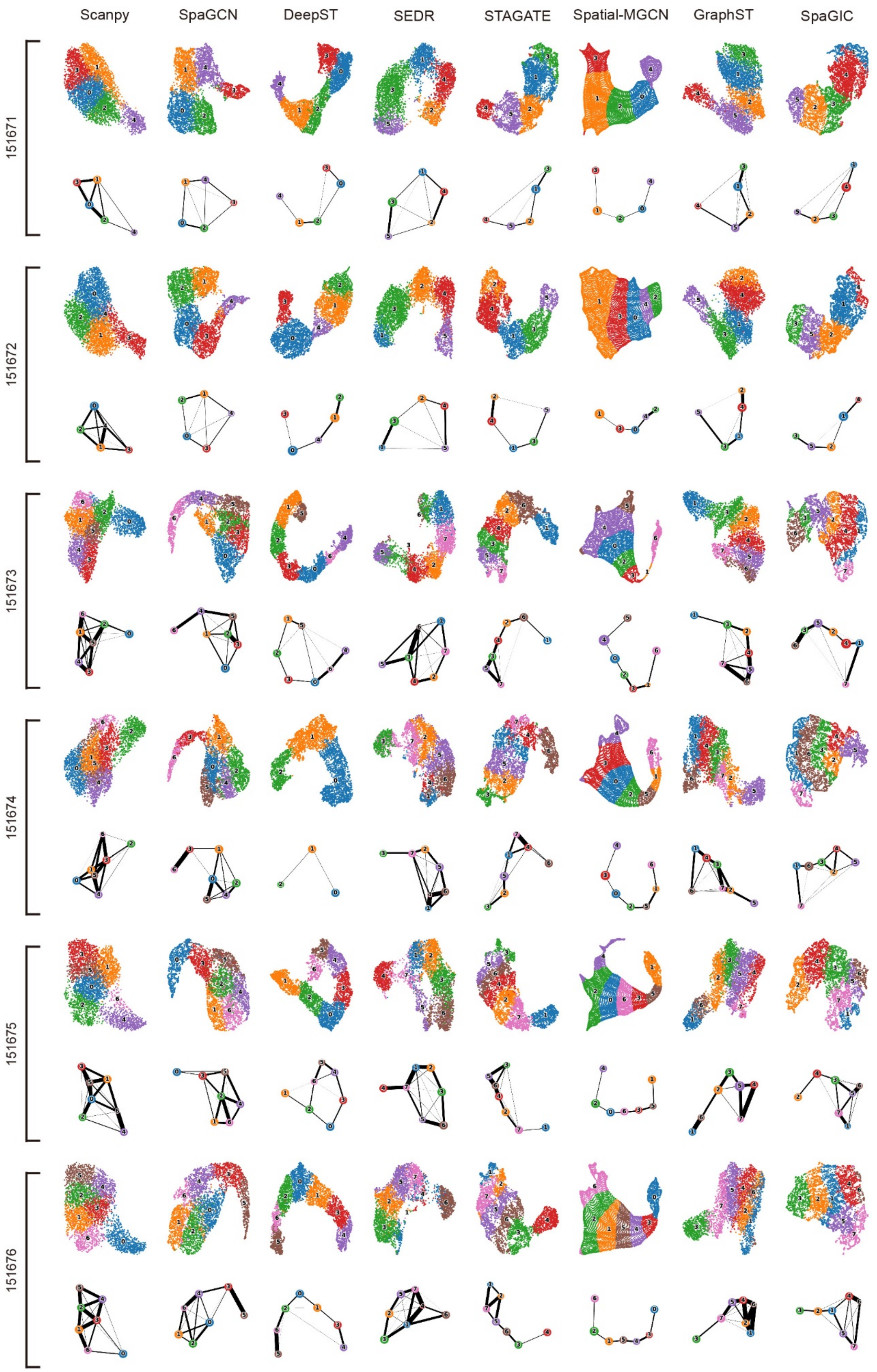

Figure S8. UMAP visualization and PAGA graphs generated by Scanpy, SpaGCN, DeepST, SEDR, STAGATE, Spatial-MGCN, GraphST and SpaGIC embeddings respectively.

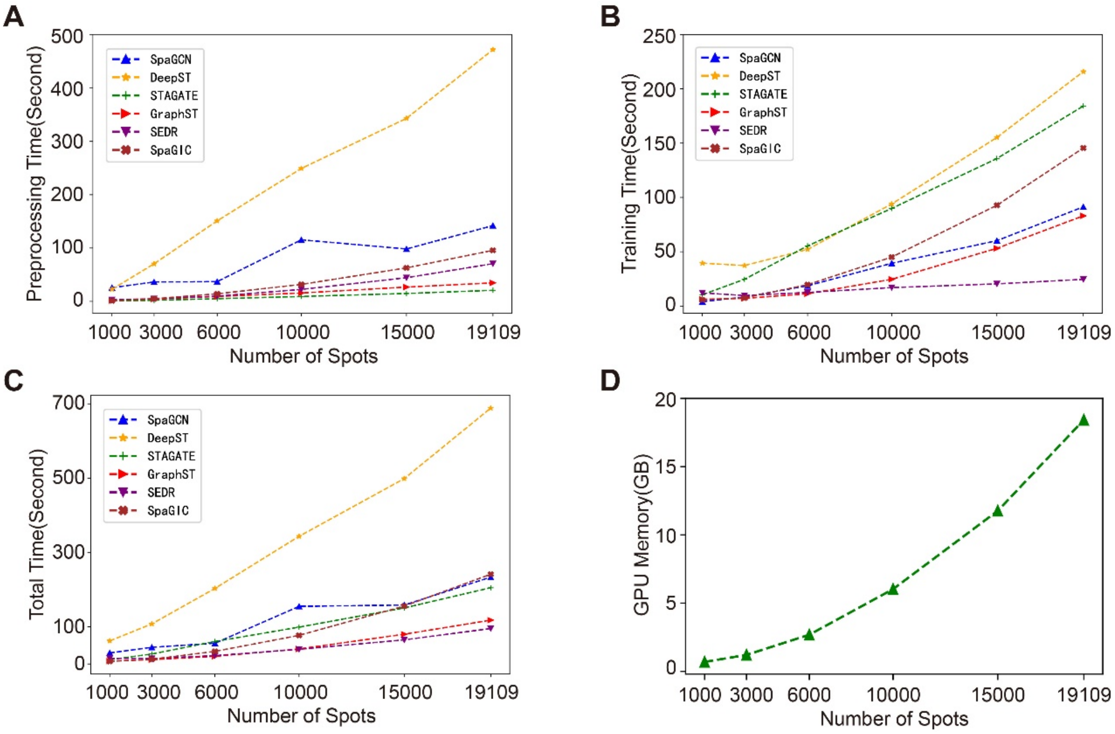

Figure S9. Running time and GPU memory usage on the randomly sampled datasets derived from the Stereo-seq MOB dataset, which includes different numbers of spots, based on a server equipped with an Intel(R) Xeon(R) Silver 4208 CPU @ 2.10GHz and an NVIDIA GeForce RTX 4090 GPU. (**A-C**) Preprocessing time, training time, total time, respectively. (**D**) GPU memory usage.

Supplementary Tables

Table S1. Summary of all datasets used in this study.

Platform	Tissue	Section	Spots/Bins	Genes
10x Visium	Human dorsolateral prefrontal cortex (DLPFC)	151507	4226	33538
		151508	4384	
		151509	4789	
		151510	4634	
		151669	3661	
		151670	3498	
		151671	4110	
		151672	4015	
		151673	3639	
		151674	3673	
		151675	3592	
		151676	3460	
	Human breast cancer	Section 1 V1.1.0	3798	36601
	Mouse brain anterior	Section 1 V1.1.0	2695	32285
Stereo-seq	Mouse olfactory bulb	-	19109	27106
Slide-seqV2	Mouse olfactory bulb	Puck_200127_15	21724	21220
STARmap	Mouse visual cortex	-	1207	1020
osmFISH	Mouse somatosensory cortex	-	3405	33

Table S2. ARI and NMI scores of Scanpy, SpaGCN, DeepST, SEDR, STAGATE, Spatial-MGCN, GraphST and SpaGIC on the DLPFC dataset.

Section	SpaGIC		GraphST		Spatial-MGCN		STAGATE		SEDR		DeepST		SpaGCN		Scanpy	
	ARI	NMI	ARI	NMI	ARI	NMI	ARI	NMI	ARI	NMI	ARI	NMI	ARI	NMI	ARI	NMI
151507	0.607	0.683	0.431	0.648	**0.645**	**0.751**	0.539	0.654	0.571	0.698	0.485	0.619	0.461	0.584	0.354	0.411
151508	0.487	0.610	**0.500**	**0.647**	0.462	0.602	0.495	0.618	0.462	0.616	0.405	0.529	0.364	0.463	0.307	0.387
151509	**0.572**	0.675	0.428	0.627	0.550	**0.684**	0.513	0.630	0.428	0.609	0.497	0.647	0.451	0.594	0.373	0.452
151510	**0.550**	0.644	0.469	0.636	0.517	**0.668**	0.423	0.605	0.508	0.655	0.458	0.598	0.444	0.579	0.319	0.391
151669	0.483	0.598	**0.588**	**0.650**	0.390	0.501	0.481	0.607	0.394	0.587	0.450	0.538	0.262	0.395	0.305	0.393
151670	0.501	0.575	**0.682**	**0.681**	0.357	0.526	0.435	0.573	0.541	0.565	0.303	0.457	0.365	0.480	0.257	0.336
151671	**0.688**	**0.750**	0.609	0.721	0.602	0.708	0.594	0.679	0.620	0.699	0.500	0.651	0.537	0.649	0.346	0.432
151672	0.679	0.726	0.620	0.719	**0.774**	**0.752**	0.593	0.684	0.631	0.697	0.467	0.603	0.568	0.663	0.343	0.422
151673	0.611	0.717	**0.635**	**0.734**	0.599	0.660	0.594	0.703	0.514	0.662	0.545	0.699	0.422	0.575	0.376	0.493
151674	**0.622**	**0.721**	0.580	0.705	0.596	0.690	0.604	0.719	0.558	0.674	0.462	0.620	0.391	0.554	0.362	0.425
151675	0.505	0.612	0.545	0.670	0.545	0.665	**0.658**	**0.740**	0.572	0.707	0.638	0.718	0.466	0.579	0.318	0.411
151676	**0.606**	0.686	0.585	0.665	0.576	0.673	0.579	0.687	0.534	0.664	0.569	**0.688**	0.325	0.511	0.297	0.392
Median	**0.589**	**0.679**	0.583	0.667	0.563	0.671	0.559	0.667	0.538	0.663	0.476	0.620	0.433	0.577	0.331	0.411
Mean	**0.576**	0.666	0.556	**0.675**	0.551	0.657	0.542	0.658	0.528	0.653	0.482	0.614	0.421	0.552	0.330	0.412
Variance	0.072	0.058	0.082	0.036	0.112	0.078	0.073	0.052	0.072	0.048	0.083	0.076	0.086	0.077	0.035	0.039
